# Comprehensive characterization of a time-course transcriptional response induced by autotoxins in *Panax ginseng* using RNA-Seq

**DOI:** 10.1186/s12864-015-2151-7

**Published:** 2015-11-25

**Authors:** Bin Wu, Qiliang Long, Yuan Gao, Zi Wang, Tianwei Shao, Yanan Liu, Yong Li, Wanlong Ding

**Affiliations:** Institute of Medicinal Plant Development, Chinese Academy of Medical Sciences & Peking Union Medical College, Beijing, China

**Keywords:** RNA-Seq, *Panax ginseng*, Autotoxicity, Benzoic acid

## Abstract

**Background:**

As a valuable medicinal plant, the yield of *Panax ginseng* is seriously affected by autotoxicity, which is a common phenomenon due to continuous cropping. However, the mechanism of autotoxicity in *P. ginseng* is still unknown.

**Results:**

In total, high throughput sequencing of 18 RNA-Seq libraries produced 996,000 000 100-nt reads that were assembled into 72,732 contigs. Compared with control, 3697 and 2828 genes were significantly up- and down-regulated across different tissues and time points, respectively. Gene Ontology enrichment analysis showed that ‘enzyme inhibitor activity’, ‘carboxylesterase activity’, ‘pectinesterase activity’, ‘centrosome cycle and duplication’ and ‘mitotic spindle elongation’ were enriched for the up-regulated genes. Transcription factors including AP2s/ERFs, MYBs, and WRKYs were up-regulated in roots after benzoic acid treatment. Moreover, reactive oxygen species, peroxidases and superoxide dismutase contigs were up-regulated in roots after benzoic acid treatment. Physiological and biochemical indexes showed that the proline and malondialdehyde content were restored to lower levels at a later stage after benzoic acid treatment. Benzoic acid inhibited the root hair development in a dose-dependent manner, and several differential expressed genes potentially involved in hair development were identified. Several key contigs in the flavonoid and ginsenoside biosynthesis pathways were repressed. Finally, 58,518 alternative splicing (AS) events from 12,950 genes were found after benzoic acid treatment. Interestingly, contigs in the ginsenoside biosynthetic pathway underwent AS, providing useful information about post-transcriptional regulation in *P. ginseng*.

**Conclusions:**

This study revealed the stress-response molecular mechanisms in *P. ginseng* induced by benzoic acid.

**Electronic supplementary material:**

The online version of this article (doi:10.1186/s12864-015-2151-7) contains supplementary material, which is available to authorized users.

## Background

Autotoxicity indicates that one plant releases allelochemicals into the adjacent environment, which directly or indirectly inhibit their own normal growth or that of their relatives in continuous cropping or short rotation [1–3]. Autotoxicity commonly occurs both in nature and cultivation and affects the yield and quality of plants [4–6]. The phenomenon was thought to be caused by soil-borne pathogens that gradually accumulated during continuous cropping at first. However, evidence showed that secondary micromolecular metabolites released by plants are important factors [2]. Autotoxicity has been found in many plants, such as rice [7], wheat [8], sweet potato [9], apple [10] and alfalfa [11]. Recently, medicinal plants were also reported to exhibit autotoxic phenomena [12–14]. Autotoxicity has been reported in more than 50 plant species thus far [2, 15].

Potential autotoxins have been explored for different compounds. For example, water-soluble organic acids, aliphatic aldehydes, lactones, long-chain fatty acids, naphthoquinones, anthraquinones, coumarins, tannins, steroids, alkaloids, cyanohydrins, sulfides, oil glycosides and purines have been isolated and identified as allelochemicals [2, 4]. Other types of autotoxins including phenolic compounds, flavones and terpenoids, and phenolic compounds are the main types of autotoxins [2, 4]. The main phenolic acids are ferulic acid, o-hydroxy phenyl acetic acid, p-coumaric acid, ferulic acid, iso-ferulic acid, malic, critic, fumatic, and caffeic acids [16–18]. Microarrays have been used to investigate the gene expression profiling in plants in response to allelochemicals such as 2(3H)-benzoxazolinone [19], gallic acid [20], 3-(3’,4’-dihydroxyphenyl)-L-alanine [21], juglone [7] and ferulic acid [22]. However, DNA microarrays have limitations including background hybridization and known probe information. The recently developed next generation high throughput RNA sequencing (RNA-Seq) technique directly sequences transcripts to obtain a large dynamic range of expression levels and may provide comprehensive clues to solve this problem [23]. Furthermore, RNA-Seq can be used directly for transcriptome profiling for species with [24] or without [25] available genomes. Therefore, RNA-Seq has potential to replace microarray technology [26].

*Panax ginseng* is a highly valuable perennial herb with medicinal properties that is native to China and Korea [27] . However, continuous cropping of *P. ginseng* results in autotoxicity and a decline in biomass. To date, transcriptome studies of *P. ginseng* have focused on ginsenoside biosynthetic genes [28–31]. Transcriptome studies of *P. ginseng* after autotoxin treatment have not been reported. Benzoic acid is one of the major autotoxins identified in *P. ginseng* root exudates and rhizosphere soil. It significantly inhibited seed germination and growth [32, 33]. Although the autotoxins in *P. ginseng* have been isolated and identified, their mechanisms remain unknown. In this study, we constructed RNA-Seq libraries using RNA extracted from roots, stems, and leaves treated with benzoic acid at six different time points and revealed enriched functional terms in response to this stress. Interestingly, several transcript factors were up-regulated in roots, suggesting the importance of transcription factors in response to benzoic acid. Moreover, peroxidase (POD) and superoxide dismutase (SOD) response to benzoic acid induction were identified. Several key contigs involved in the flavonoid and ginsenoside biosynthesis pathways were repressed. These results provide a comprehensive understanding of the *P. ginseng* response to benzoic acid stress and lay a foundation for improving the resistance or endurance of *P. ginseng* to autotoxins in the environment.

## Results

### RNA sequencing and *de novo* assembly

RNA samples were collected from roots, stems and leaves at 0 days post treatment (DPT), 1 DPT, 3 DPT, 5 DPT, 7 DPT and 9 DPT after benzoic acid treatment. Then, RNA-Seq was performed to investigate DEGs or pathway responses to benzoic acid. In total, 18 RNA-Seq libraries generated approximately 996,000 000 clean reads of 100 nt in length (Table 1). All the sequencing reads are deposited in the NCBI short read archive (SRA) under the accession number SRP049125. The reads were pooled together and assembled into reference sequences, yielding 72,732 contigs representing 272,053,772 total assembled bases for the *P. ginseng* transcriptome. The GC content was 38.55 %. The N50 and average length of assembled sequences were 1794 bp and 1428 bp, respectively (Additional file 1). This transcriptome assembly project has been deposited in DDBJ/EMBL/GenBank under the accession GDQW00000000.

**Table 1:** Summary of Illumina sequencing and transcriptome assemblies for RNA-Seq libraries

Indcued time	Tissues	Reads	Length	Total bases	Mapping reads	Unique reads	Unigenes	SRA experiment IDs
0 day	root	54,802,994	100,100	5,480,299,400	46,616,140	38,539,562	19,524	SRX758350
	stem	62,654,934	100,100	6,265,493,400	53,955,686	44,640,828	19,207	SRX758356
	leaf	62,304,990	100,100	6,230,499,000	53,976,979	45,581,048	18,886	SRX758362
1 day	root	53,367,552	100,100	5,336,755,200	44,893,957	36,983,148	19,948	SRX758351
	stem	49,148,806	100,100	4,914,880,600	42,382,932	35,029,638	19,317	SRX758357
	leaf	63,531,810	100,100	6,353,181,000	54,699,654	45,625,544	19,328	SRX758363
3 day	root	54,362,660	100,100	5,436,266,000	45,401,558	37,587,578	21,180	SRX758352
	stem	48,114,866	100,100	4,811,486,600	41,208,427	33,669,930	19,900	SRX758358
	leaf	55,411,400	100,100	5,541,140,000	47,776,074	39,877,716	20,088	SRX758364
5 day	root	55,644,310	100,100	5,564,431,000	46,703,315	38,601,890	20,389	SRX758353
	stem	60,981,712	100,100	6,098,171,200	52,263,645	43,831,390	21,019	SRX758359
	leaf	54,073,298	100,100	5,407,329,800	45,762,381	37,334,730	19,633	SRX758365
7 day	root	49,978,570	100,100	4,997,857,000	41,697,612	34,220,122	20,354	SRX758354
	stem	52,655,072	100,100	5,265,507,200	45,398,187	37,654,496	18,971	SRX758360
	leaf	56,194,980	100,100	5,619,498,000	48,170,802	40,207,756	18,385	SRX758366
9 day	root	55,412,038	100,100	5,541,203,800	46,873,595	38,947,342	19,271	SRX758355
	stem	53,283,108	100,100	5,328,310,800	46,033,892	37,871,864	17,644	SRX758361
	leaf	54,119,488	100,100	5,411,948,800	46,471,434	38,712,836	18,776	SRX758367

### Annotation of the ginseng transcriptome

The assembled transcripts were searched against the sequences in the NT database using the BLASTN algorithm (E-value < 10^−6^) for functional annotations. To obtain comprehensive annotation, coding regions were extracted using Trinity software [34]. These protein sequences were searched against the UniProt and NR databases using the BLASTP algorithm (E-value < 10^−6^). Complete annotation information was listed in Additional file 2. A total of 11,838, 15,469, and 21,807 genes were annotated in the UniProt, NR and NT databases, respectively. Finally, 28,139 genes were annotated, and the NT database had the largest match, followed by the NR and UniProt databases. BLAST was also performed against the KEGG database to annotate the metabolic pathways for each gene. A total of 2783 genes in 262 pathways were identified according to the KEGG database (Additional file 3).

### Calculation of gene expression

Gene expression was measured by mapping RNA-Seq reads from 18 libraries to the assembled sequences. On average, 85 % of total reads were successfully mapped to the assembled transcript sequences using Bowtie2 2.2.3 [35]. Subsequently, FPKM was adopted to quantify the expression of 72,732 contigs*.* Both pairs of PE reads with unique location were retained to calculate FPKM value in the following analysis to detect the DEGs associated with benzoic acid stress. There were 704,917,418 (71 % of the total) reads that were mapped back in pairs with unique locations on the assembled genes and used for the downstream FPKM calculation. The average FPKM value for all samples examined was 26. Additional file 4 shows the distributions of FPKM values followed by log2 transformation. In total, 26,973 genes with the minimum expression threshold of FPKM > 3 were identified in at least one RNA-Seq library.

### Identification of DEGs and dynamic profile

**Fig. 1:**
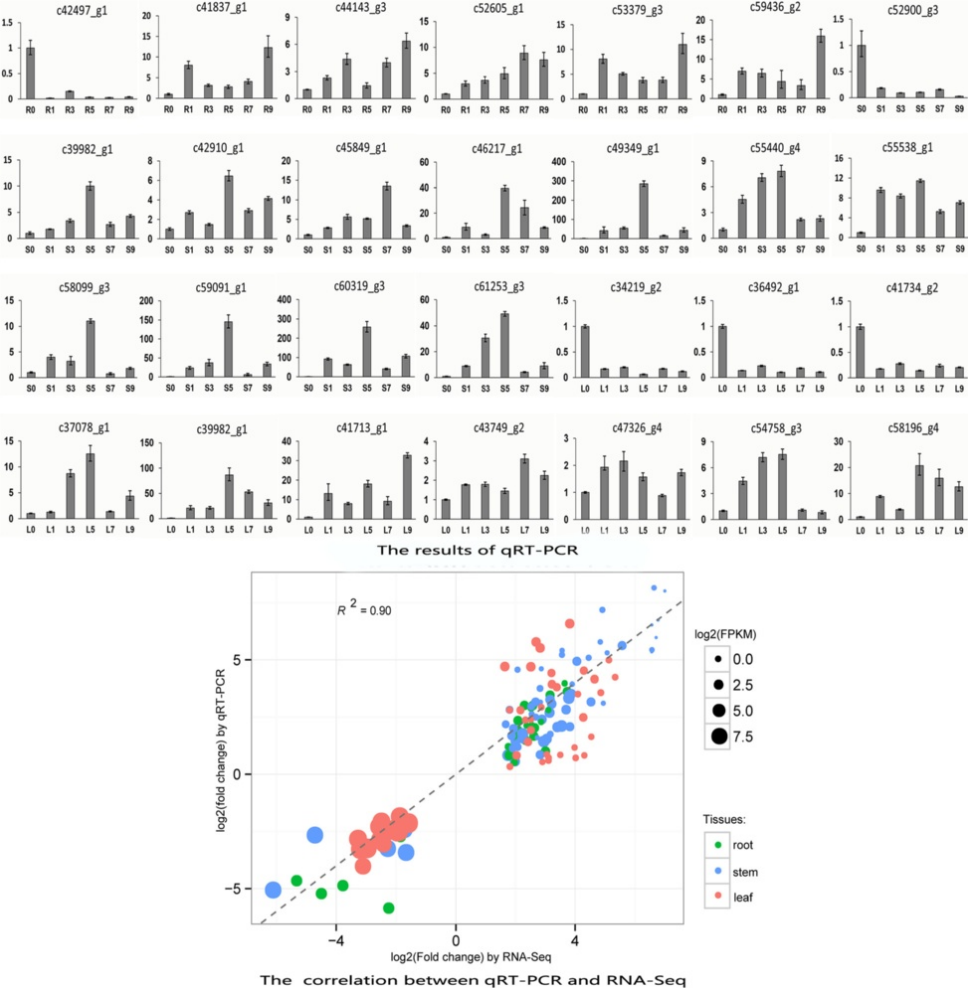
Expression patterns of 28 randomly selected DEGs by qRT-PCR and the correlation between qRT-PCR and RNA-Seq. Fold changes of the transcript levels at different time points are shown. The average expression level in 0 DPT was set to 1. Error bars represent standard error. L, S and R represent leaves, stems and roots, respectively. The X-axis represents log2 fold change of the qRT-PCR. The Y-axis indicates the log2 value of the fold change from RNA-Seq. The size of each point is proportional to the log2 (FPKM) in 0 DPT. Leaves, stems and roots were represented by points with different color

**Fig. 2:**
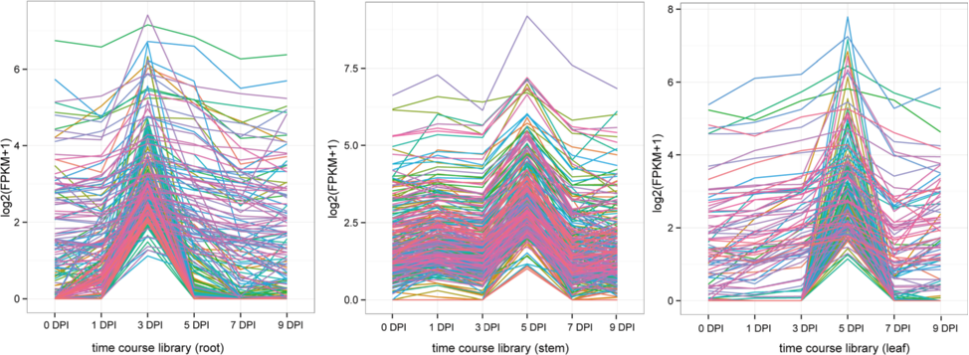
Representative time-course profile clusters. Representative profile clusters for roots, stems and leaves. For each time point and each gene, the log2 (FPKM + 1) is shown for the mean expression profile for each cluster

Each DEG was evaluated by the MA-plot-based method with random sampling model [36] to calculate the *P* value by the pair-wise comparison of control samples and benzoic acid induced samples. The stringent cut-offs for DEGs included a three-fold or higher change and *P* < 0.001. A total of 3697 and 2828 genes were identified with significantly up- and down-regulated expression, respectively, compared with control (Table 2). All the differential expressed genes could be found at Additional file 5. To validate the RNA-Seq results, qRT-PCR was carried out on 28 randomly chosen DEGs at 0 DPT, 1 DPT, 3 DPT, 5 DPT, 7 DPT and 9 DPT. The expression profiles based on RNA-Seq could be found at Additional file 6. The qRT-PCR results were highly consistent with those from the RNA-Seq (Fig. 1). The Pearson correlation coefficient was 0.90, indicating that the qRT-PCR results were positively linearly related with the RNA-Seq results (Fig. 1). The RNA-Seq profiles of 28 randomly chosen DEGs and all the genes that described in the result parts were validated by qRT-PCR (Additional files 7, 8, 9, 10, 11 and 12). Genes with similar expression patterns might be functionally correlated [37]. In order to reveal the expression change after benzoic acid treatment, six time points and three tissues across 18 RNA-Seq libraries were used to identify statistically significant expression profiles and the genes associated with the profiles. To investigate the dynamic patterns of these DEGs after benzoic acid treatment, gene expression cluster analysis was performed using the STEM algorithms [38]. In brief, the STEM algorithm first selects a set of representative model profiles. Then the correlation coefficient for each gene was used to identify the most closely model profile. Then the profiles with statistically significant higher number of genes were selected using permutation test. STEM algorithm analyses of the expressed genes generated three significant clusters (*P* < 0.001) based on the expression profile similarity. Cluster 1 included 263 genes which increased sharply at early time points (3 DPT) after benzoic acid treatment in roots (Fig. 2). Genes in this cluster were predominantly enriched in response to toxin metabolic process (3.4398E-4) and response to stress (3.9015E-3) based on GO analysis. However, for cluster 2 in the stems and cluster 3 in the leaves, the maximum abundance shifted to later time points (5 DPT) (Fig. 2). The gene numbers for these two clusters were 304 and 168, respectively. GO terms enriched in cluster 3 also included response to stress (7.0496E-4) and response to stimulus (1.3493E-3), suggesting the importance of the genes that response to stress after benzoic acid treatment. Within contrast to these two clusters, genes in cluster 2 were enriched in nuclease activity (3.4498E-6) and DNA metabolic process (8.3423E-5), respectively.

**Table 2:** The number of up- and down-regulated DEGs based on pair-wise comparison with 0 day control (fold change >3; *P* <0.001)

Indcued time	Tissue	Up-regulated	Down-regulated
1 day	root	104	39
	stem	63	41
	leaf	139	69
3 day	root	589	41
	stem	178	230
	leaf	68	72
5 day	root	328	55
	stem	1443	751
	leaf	536	264
7 day	root	204	61
	stem	189	663
	leaf	310	242
9 day	root	785	212
	stem	490	1698
	leaf	444	634

**Fig. 3:**
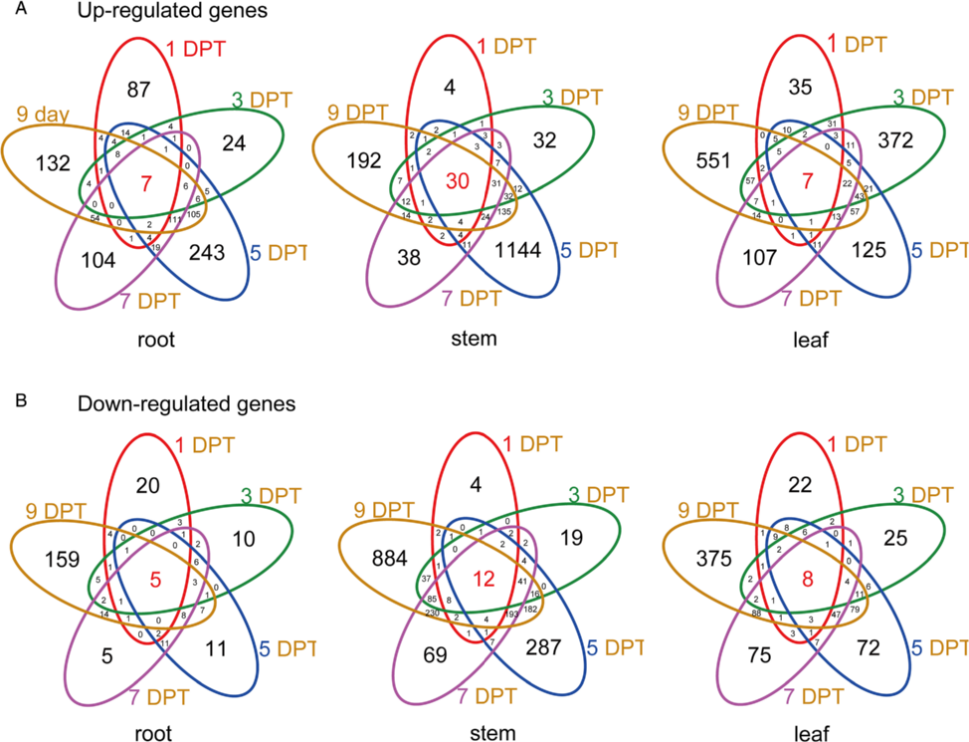
Venn diagram illustrating the overlap of the DEGs

### DEGs overlap at six different time points

DEGs that overlapped at all time points were genes induced by benzoic acid across the entire stress process. In total, seven contigs were induced across all time points (1 DPT, 3 DPT, 5 DPT, 7 DPT and 9 DPT) in roots (Fig. 3). Among these, the EG45-like domain containing protein (c28773_g1) was significantly up-regulated in roots. The qRT-PCR validation for the gene was consistent with the result of RNA-Seq (Additional file 7).

In this study, 30 contigs including five transcription factors were up-regulated across all time points in stems (Fig. 3). The function of these transcription factors was deduced from the homology protein of UniProt database in other species. Two MYB transcription factors (c49349_g1 and c59091_g1) can respond to ethylene, jasmonic acid and abscisic acid. Two No Apical Meristem (NAM) transcription factors (c53886_g5 and c48053_g1) functioned in multidimensional cell growth and the negative regulation of the abscisic acid activated signaling pathway. Transcription factor WRKY (c46217_g1) played a critical role in the response to chitin. The expression profiles based RNA-Seq and qRT-PCR validation for the above-mentioned transcript factors could be found at Additional file 7.

In the leaves, seven genes were up-regulated across all the time points (Fig. 3). The plant invertase/pectin methylesterase inhibitor has been reported as a stress-response gene [39]. In this study, we also found that one plant invertase/pectin methylesterase inhibitor (c41713_g1) gene was up-regulated in the leaves (Additional file 7).

We also observed down-regulated genes across all the time points. For example, one unigene that coded for zinc-binding dehydrogenase (c8930_g1) was down-regulated in roots, and another unigene that coded for calcium-binding protein with EF-hand domain (c41722_g1) was down-regulated in stems (Additional file 7). These results are consistent with previous reports that the lipophilicity of benzoic and cinnamic acids could impact the ion uptake and root elongation in cucumber [40]. All the differentially expressed genes at all time points could be found at Additional file 13.

### GO functional enrichment analyses

**Fig. 4:**
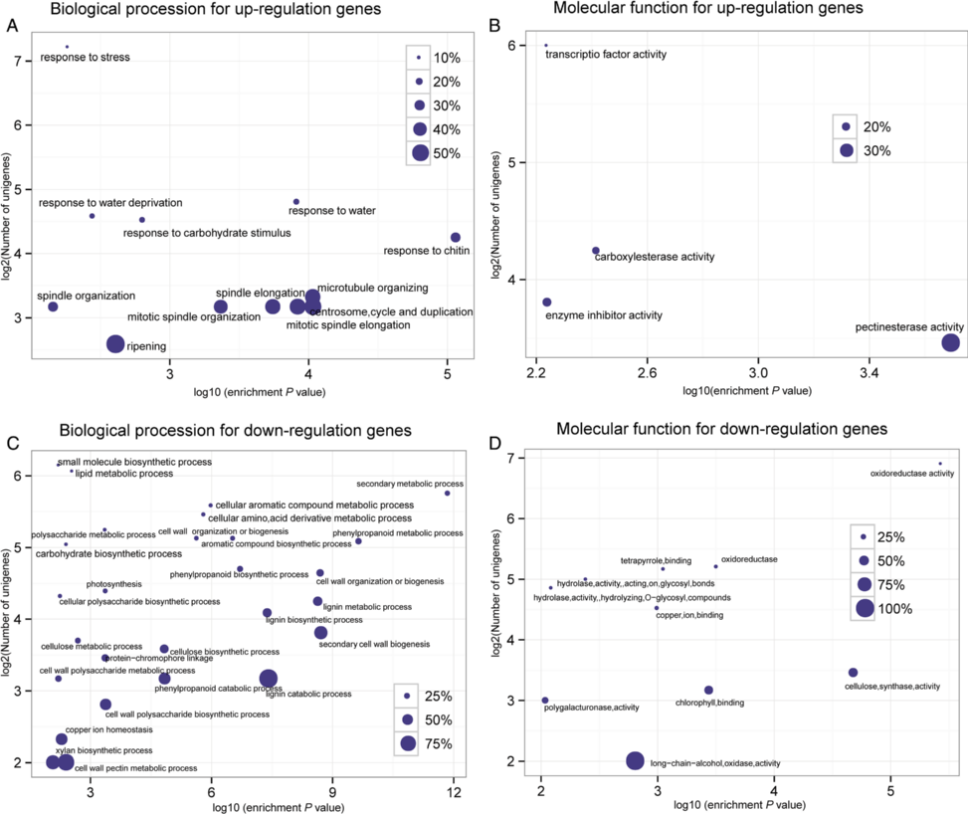
Most significantly enriched GO terms (*P* < 0.05) of the DEGs. The most significant GO terms (*P* < 0.01) are presented graphically. The X-axis represents the log10 of the enrichment *P* value. The Y-axis indicates the number of DEGs in log2 value. The size of each point is proportional to the percentage (DEGs associated with GO terms/all genes associated with GO terms). **a** and **b** presents the enriched GO term for up-regulation genes based on biological procession and molecular function, respectively. **c** and **d** presents the enriched GO term for Down-regulation genes based on biological procession and molecular function, respectively

The function of the 3697 up-regulated DEGs was determined by GO enrichment analysis. The results showed that the up-regulated genes were enriched for ‘transcription factor activity’, ‘carboxylesterase activity’, and ‘pectinesterase activity’ (Fig. 4). Up-regulated genes were also involved in ‘centrosome cycle and duplication’ and ‘mitotic spindle elongation’. Other enriched terms included ‘response to water deprivation’ (*P* = 3.6799E-3), consistent with previous findings that exposure of plant roots to ferulic acid can affect water usage [41]. We also found that ‘polysaccharide metabolic process’ (*P* = 4.5829E-4) and ‘lignin metabolic process’ (*P* = 2.5213E-9) were enriched.

Among the down-regulated genes, GO terms associated with ‘cell wall organization or biogenesis’, ‘stress response’, ‘secondary metabolic processes’ and ‘ion binding’ were enriched (Fig. 4). Down-regulated genes involved in ‘polysaccharide metabolic process’ (34 genes) and ‘lignin metabolic process’ (19 genes) showed significant responses to benzoic acid. Autotoxin usually led to a 20-50 % decrease in growth rate [2]. The photosystem is an important factor that influences plant growth. Contigs involved in ‘photosystem’ (*P* = 3.8496E-4) were significantly repressed, which can partly explain the reduction of production due to autotoxicity.

### Reactive oxygen species

Reactive oxygen species (ROS) produced from various metabolic pathways can cause oxidative damage [42]. For example, ferulic acid can produce oxidative stress in rice [22]. Plants can produce reactive oxygen species (ROS) during abiotic stress as byproducts of aerobic metabolism. At the same time plant must have the ability to remove the toxin. Benzoic acid treatment can be regarded as abiotic stress, thus the investigation of the generation and removal of ROS can help us to understand the possible function of ROS upon benzoic acid treatment.

In this study, 35 ROS-related genes were annotated as ‘response to reactive oxygen species’ (GO: 0000302 and GO: 0072593). Among them, three contigs (c14304_g1, c42911_g1 and c34265_g1) were significantly up-regulated, suggesting that these genes might take part in the oxidative damage response upon benzoic acid induction. In leaves, c14304_g1 was up-regulated at an early stage (1 DPT). However, c42911_g1 and c34265_g1 were up-regulated at a later stage in roots (9 DPT). The RNA-Seq profiles and qRT-PCR validations for the above three ROS-related genes could be found at Additional file 8.

We also investigate the differential expression levels of contigs coding POD and SOD. In total, we identified 64 contigs with peroxidase activity. Two PODs (c50418_g2 and c52462_g1) were up-regulated at 9 DPT in roots. We also identified nine contigs with SOD activity. Among them, c41673_g1 was up-regulated in roots (3 DPT) and leaves (5 DPT). The RNA-Seq profiles and qRT-PCR validations for two PODs genes (c50418_g2 and c52462_g1) and one SOD genes (c41673_g1) could be found at Additional file 8.

### Measurement of physiological and biochemical indexes of proline and malondialdehyde

**Fig. 5:**
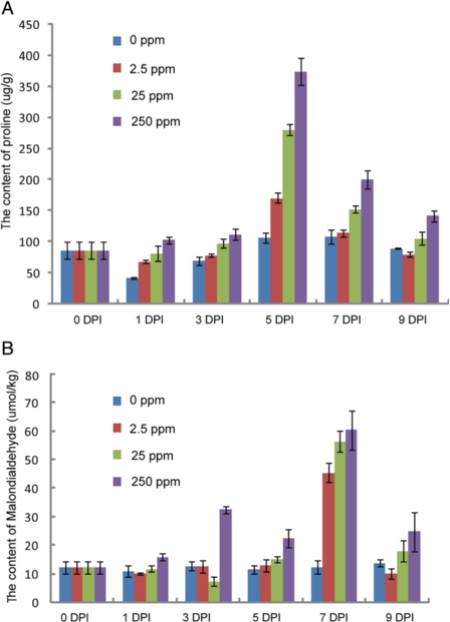
Measurement of physiological and biochemical indexes of proline (**a**) and malondialdehyde (**b**)

The impacts of benzoic acid over a concentration gradient of 2.5-250 mg · L^−1^ on the accumulation of proline and malondialdehyde (MDA) was investigated. MDA is the product of lipid membrane peroxidation and is a marker of stress-induced damage [43]. In general, the content of both free proline and MDA increased as the concentration of benzoic acid increased compared with control (Fig. 5). The accumulation of proline at 5 DPT was higher than at 0, 1, 3, 7 and 9 DPT. In the proline biosynthetic pathway, pyrroline-5-carboxylate synthetase (P5CS) and pyrroline-5-carboxylate reductase (P5CR) were two important enzymes with important roles in turning glutamic acid into proline [44, 45]. The expression level of P5CS (c61458_g1) showed a similar profile as proline. However, the expression level of P5CR (c61356_g1) was unchanged during benzoic acid treatment. For MDA (an index of lipid peroxidation), the content at 7 DPT was higher than at 0, 1, 3, 5 and 9 DPT, suggesting that benzoic acid treatment promoted lipid peroxidation at later stage. Ascorbate is catalyzed and converted into MDA by ascorbate peroxidase (APX) [46]. According to the RNA-Seq results, the expression of APX (c49700_g1) increased at the early stage and decreased at the later stage, consistent with the change in MDA content. The RNA-Seq profiles for P5CS, P5CR and APX, together with the qRT-PCR validations were shown at Additional file 9.

### Effect of autotoxicity on root hair development

**Fig. 6:**
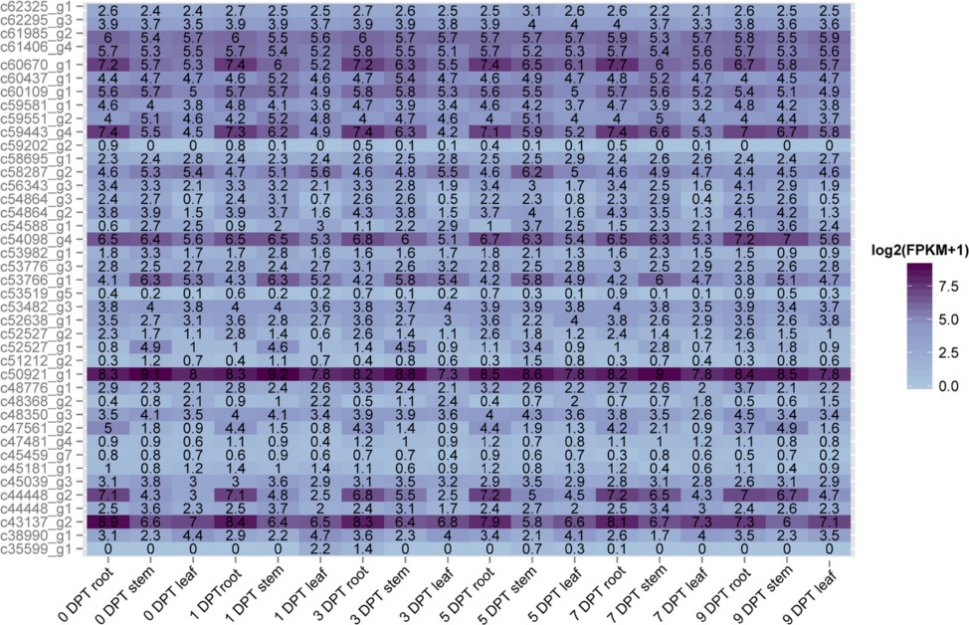
Plot of the 41 root hair related genes according to FPKM values

Autotoxins produced by plants inhibit their own growth directly or indirectly. For example, ferulic acid inhibited root elongation in rice [4]. Therefore, we also observed the root morphology after benzoic acid treatment. The results showed that benzoic acid caused permanent morphological reductions in root hair density by observation of root morphology using stereomicroscope 4. The adverse effect of autotoxins on root development increased as the concentration of benzoic acid increased (Additional file 14). The root hair growth was almost completely inhibited when the concentration of benzoic acid reached 250 mg · L^−1^, suggesting that benzoic acid repressed the root hair in a concentration-dependent manner. The possible mechanism of the effect of benzoic acid on root hair formation was investigated. In total, 41 root hair related contigs were identified from the homology protein of UniProt database in other species, and the contigs (c60670_g1, c59443_g4, c47561_g2, c44448_g2 and c43137_g2) were highly expressed in roots compared with leaves and stems (Fig. 6). Among these root hair related contigs, four up-regulated (c35599_g1, c44448_g2, c47561_g2 and c54588_g1) and two down-regulated (c52527_g1 and c53982_g1) contigs might contribute to the low density of root hairs upon benzoic acid treatment. c35599_g1 was up-regulated at 3 DPT in roots; both c44448_g2 and c47561_g2 were up-regulated in stems (7 DPT and 9 DPT) and c54588_g1 was up-regulated in roots (9 DPT). Two down-regulated contigs were observed in stems. The qRT-PCR validations with RNA-Seq profiles for the above nine genes could be found at Additional file 10.

### Flavonoid and ginsenoside biosynthetic pathways upon benzoic acid treatment

Previous studies revealed that flavonoids were also involved in autotoxicity and resulted in root growth inhibition and cell division reduction in root meristematic regions [47]. Chalcone synthase (CHS), chalcone isomerase (CHI), flavanone 3-hydroxylase (F3H), flavonoid 3’ 5’-hydroxylase (F3’5’H), flavonoid 3’-hydroxylase (F3’H), anthocyanidin synthase (ANS), and UDP-glucose:anthocyanidin 3-O-glucosyltransferase (A3GT) genes are key enzymes in the flavonoid biosynthesis pathway [48, 49]. In this study, we identified one CHS unigene (c57094_g1), two CHI contigs (c366_g1 and c45255_g1), one F3H contigs (c42137_g1) and two A3GT contigs (c43015_g1 and c63923_g1). Interestingly, CHI (c45255_g1) was repressed in stems at the later stage (5 DPT and 7 DPT) after benzoic acid treatment. F3H (c42137_g1) was also repressed in roots at the later stage (7 DPT and 9 DPT). The RNA-Seq profiles and qRT-PCR validations for CHI and F3H could be found at Additional file 11.

Ginsenosides are the major bioactive components of *P. ginseng.* Due to their important pharmacological effects, the ginsenoside biosynthetic pathway has been widely investigated [28–31]. Cytochrome P450 involved in the ginsenoside biosynthetic pathway might represent the largest family and catalyze most of the oxidation steps during the biogenesis of plant secondary metabolism. In this study, 20 potential cytochrome P450 contigs were down-regulated and 13 were up-regulated. The remaining contigs involved in the ginsenoside biosynthetic pathway were down-regulated upon benzoic acid treatment. The down-regulated DEGs included three farnesyl diphosphates (FPS), two-geranylgeranyl pyrophosphate synthase (GPS), one hydroxymethylglutaryl-CoA synthase (HMGS) and two UGT (UDP-glycosyltransferases). The RNA-Seq profiles and qRT-PCR validations for the two FPS (c47877_g1, c48923_g5,), two GPS (c47128_g1, c7968_g1), one HMGS (c60578_g1) and two UGT (c51692_g1, c64659_g1) for CHI and F3H could be found at Additional file 11.

### The expression of transcription factors upon benzoic acid treatment

**Fig. 7:**
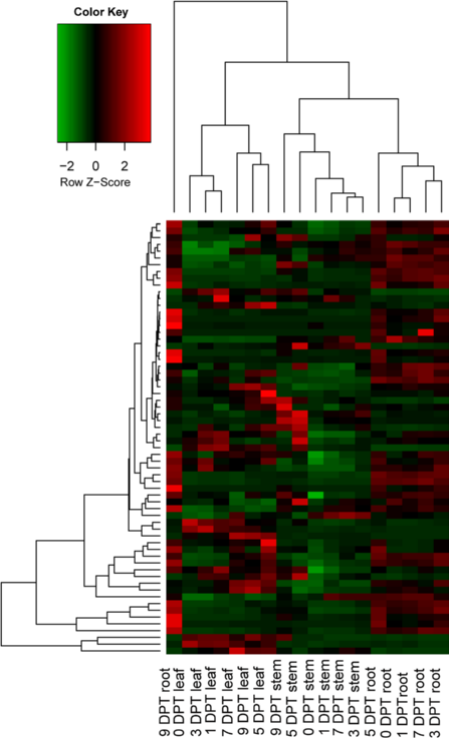
Hierarchical clustering of differentially expressed TFs from 18 libraries. Hierarchical clustering of the DEGs among roots, stems and leaves at 0 DPT, 1 DPT, 3 DPT, 5 DPT, 7 DPT and 9 DPT using FPKM expression values from RNA-Seq. Each column represents an RNA-Seq library and each row represents a DEG. Different colors shown different expression levels, with green representing low expression levels and red representing high expression levels

TFs function in the abiotic stress tolerance response, and overexpression of these TFs generated transgenic plants with significant tolerance to abiotic stresses [50–53]. A total of 64 TFs were significantly up-regulated by benzoic acid, and TF expression levels were higher in roots than in other tissues (Fig. 7). In total, 10 % of TFs in the TF family were up-regulated in roots (*P* = 5.8375E-3). Our results revealed that the expression of three AP2/ERF (c46110_g2, c46110_g3), four MYB (c34519_g1, c49349_g1, c55923_g2, c59091_g1), and nine WRKY genes (c46449_g1, c49987_g2, c49987_g1, c50394_g2, c51336_g1, c54471_g1, c55588_g2, c59948_g3, c46217_g1) were up-regulated in response to benzoic acid, suggesting that responses to benzoic acid might require the up-regulated TFs in roots to regulate downstream response genes. The qRT-PCR validations and RNA-Seq profiles for these TFs could be found at Additional file 12.

### Alternative splicing of the transcriptome upon benzoic acid induction

**Fig. 8:**
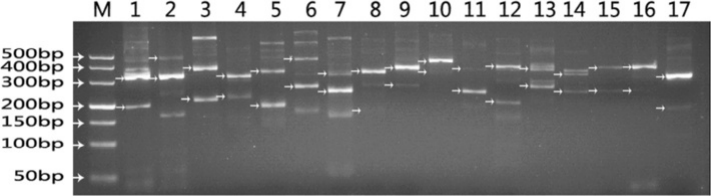
Validation of randomly selected AS events by RT-PCR

In eukaryotes, alternative splicing (AS) increases the diversity of the transcriptome and the proteome and takes part in biological process such as plant-virus interactions [54] and stress-related regulation [55]. However, the AS events upon benzoic acid treatment have never been reported. We used previous published method to identify AS events in a genome-wide way [25]. In brief, we used BLAT to compare Trinity assembled sequences with the following options: −tileSize = 18 -oneOff = 0 -minIdentity = 96 -out = sim4 -maxIntron = 10000. Then the candidates were revealed according to the pair-wise comparisons to search for indels. Finally, Indels that were flanked by two sequentially matching sections were regarded as candidate AS regions. In total, 58,518 AS events in 12,950 AS genes were found upon benzoic acid induction. AS events from 17 randomly selected genes were validated by RT-PCR. Then the expected band sizes can be predicted according to the RNA-Seq results and were listed at Additional file 15. The real band sizes from RT-PCR that matched the expected band size from RNA-Seq were arrowed in Fig. 8. For genes c15452_g1 and c11325_g1, we only observed one clear expected band, however, the other weak bands might be caused by low abundance. The other 15 genes can be detected all expected bands and the obtained production sizes of RT-PCR were consistent with that of the RNA-Seq results. Splicing factors are extensively alternatively spliced. In this study, we found 113 splicing factors. Among these splicing factors, 61 splicing factors were regulated by AS. In total, the AS splicing factor percentage was 54 %, higher than the average AS ratio (18 %). Among the contigs involved in the ginsenoside biosynthetic pathway, one AACT, one AS, 3 FPS, 1 GPS, 2 HMGR, 1 HMGS, 1 SE, 4 SS and 5 UGT contigs were regulated by AS.

## Discussion

Autotoxicity reduced both yield and quality of crops in a continuous cropping pattern [18]. *P. ginseng*, one of the most important traditional Chinese herbal medicines, exhibited a severe autotoxicity problem. In our previous study, secondary metabolites including benzoic acid were identified with strong autotoxic and growth-inhibiting activity on *P. ginseng* [33]. However, the molecular mechanism by which *P. ginseng* responds to benzoic acid remains unknown. In this study, we investigated the effect of benzoic acid on gene expression profiles using RNA-Seq technology and identified the differentially expressed genes that directly respond to autotoxins and the enriched GO terms that were involved in multiple biological processes. Moreover, we identified three up-regulated ROS-related genes. ROS can damage cell membranes and other components. The ROS-scavenging system involves three major enzymes: SOD, POD and CAT [46]. In this study, we found that SOD and POD contigs were up- regulated at later stages, which might enhance the anti-oxidation mechanisms at the later stage of benzoic acid induction. The physiological and biochemical indexes for proline and MDA also support this conclusion. Consistent with the gene expression profiles of SOD and POD, the proline and MDA content decreased at the later stage of benzoic acid treatment. Both free proline and MDA were restored to the lower level at a later stage (9 DPI), implying that *P. ginseng* partly became resistant to benzoic acid stress at the later stage.

We also found that two important contigs that coded for the enzymes CHI and F3H in the flavonoid biosynthesis pathway were repressed at the later stage. Flavonoid production might be repressed at the later stage by the down-regulation of CHI and F3H upon benzoic acid treatment. In this way, flavonoid could be presented at a relatively low concentration, and the exudation of flavonoid into soil could be reduced. The reduced concentrations of flavonoid in the soil can the release some of the adverse effects of autotoxin. In the future, the actual flavonoid concentrations upon benzoic acid treatment will be measured to obtain accurate information about the regulation of flavonoid biogenesis and transporters in the response to autotoxins.

Autotoxicity and pathogens are two major causes of continuous cropping [6, 56]. Thus, several groups have investigated the interplay between autotoxins and pathogens in soil. Autotoxins can be degraded by pathogens [57, 58] and repress the prevalence of soil pathogens [2]. However, another study showed that autotoxins exuded from the root of Ginseng saponins stimulated the growth of soil pathogens [59]. Baicalin released from *Scutellaria baicalensis* Georgi increases the activity of soil pathogens [14]. The suppression or enhancement effect of autotoxins on pathogens can be explained by the diversity of responses of plants to different autotoxins and pathogens. The accumulation of *C. destructans,* one of the most destructive pathogens, results in serious root rot disease and prevents the continuous cultivation of *P. ginseng* [60]. Autotoxins in the rhizosphere in *P. ginseng* affect and interact with pathogens in soils. However, a study on this topic has never been reported in *P. ginseng*. We still do not know the suppression or enhancement effects of autotoxins on the most destructive pathogens. In this study, we found that ‘stress response genes’ was enriched in differentially expressed genes after exposure to benzoic acid. These genes might also affect disease resistance upon *Cylindrocarpon destructans* infection. Understanding the interaction underlying autotoxins and pathogens is necessary to effectively control both *P. ginseng* root diseases and the adverse effects of autotoxicity.

Previous studies showed that the autotoxin potential of many plants is species-dependent [2]. The autotoxin was toxic to cucumber but not to fig leaf gourd plants [61]. In this study, we only uncovered the transcriptional response induced by benzoic acid. Thus, in the future it will be interesting to investigate different *P. ginseng* autotoxins to reveal the similarities and differences in the molecular mechanisms of autotoxin-induced responses.

## Conclusions

*P. ginseng* is a suitable plant for the study of autotoxicity because it is a highly valuable perennial herb and its biomass is affected by continuous cropping. Based on 18 RNA-Seq libraries from roots, stems, and leaves at different time points, we obtained transcriptome profiles and identified the DEGs upon benzoic acid stress. More importantly, changes in transcription factors upon benzoic acid induction were identified. Additionally, the study analyzed the reactive oxygen species genes and measured the physiological and biochemical indices of proline and MDA, which will help to reveal the molecular mechanisms of autotoxicity. Moreover, several differentially expressed root hair related genes that coded for key enzymes in the flavonoid biosynthesis pathway were identified, which might contribute to the dose-dependent inhibitory effect of benzoic acid on root hair development. Finally, the down-regulated contigs in the ginsenoside biosynthetic pathway were identified, and the repression of these contigs can reduce the biosynthesis of ginsenoside and partly release the adverse effects of autotoxins. This study provided a comprehensive characterization of the transcriptional response induced by autotoxins.

## Methods

### Plant growth conditions and sample collection

Roots of 3-year-old *P. ginseng* cv. Damaya were collected from the experimental field of the Institute of Medicinal Plant Development, Chinese Academy of Medical Sciences. Healthy *P. ginseng* roots were sterilized by 50 % carbendazol wettable powder (diluted 800 times) for 10 min and then rinsed thrice in distilled water. The plants were transplanted into sterilized silica sand in plastic square pots (40 cm × 20 cm × 15 cm) with a density of 48 plants/pot and placed in a greenhouse under a 16 h light, 8 h dark period at 25 °C to control soil moisture, temperature, fertility, pathogens, and other unpredictable factors that may influence disease severity and pathogen growth. Hoagland nutrient solution was used to provide essential nutrients for the normal growth of the *P. ginseng* plants [62]. In this work, when the leaves of *P. ginseng* seedlings were completely spread, benzoic acid (25 mg · L^−1^) was sprayed evenly on the sterilized silica sand with a volume of 200 mL/pot because benzoic acid with 25 mg · L^−1^ significantly reduced root hair growth. The control was sprayed with Hoagland nutrient solution without benzoic acid.

### RNA extraction, library construction and sequencing

The roots, stems and leaves of *P. ginseng* under benzoic acid stress were collected at 0 day, 1 day, 3 day, 5 day, 7 day and 9 day post treated (DPT). The samples were frozen in liquid nitrogen immediately after the surface was washed by distilled water. The tissues collected from the 0 day served as a control. In total, 18 samples were used to detect the global changes in gene expression. Total RNA was extracted using TRizol reagent according to the manufacturer’s instructions and then digested with RNase-free DNase to eliminate the contamination of genomic DNA. Finally, an Agilent Technologies 2100 Bioanalyzer was used to measure and quantify the total RNA. The RNA samples with concentrations higher than 400 ng · ul^−1^ and an RNA integrity number (RIN) > 8 were used for the RNA-Seq library construction and reverse transcription PCR (RT-PCR).

RNA-Seq libraries were constructed using Illumina’s kit following the paired-end sample preparation kit protocol. In brief, the mRNA of each sample was enriched using the oligo (dT) magnetic beads. Then, purified RNA was cleaved into short fragments (~330 nt) by adding fragmentation buffer prior to cDNA synthesis. Subsequently, the short fragments were ligated to sequencing adapters. Fragments with suitable sizes (400 ~ 500 nt) were purified by agarose gel electrophoresis and then were selected to be templates for PCR amplification to produce the library and sequence via the Illumina HiSeq™ 2000 sequencing platform. All raw reads were pair-end sequenced and subjected to the following quality control to produce clean reads: (1) raw reads including adapter sequences and empty adapter were discarded; (2) reads including unknown N bases comprising more than 3 % of the total length were filtered; (3) reads including low-quality bases that comprise more than 15 % of total length were discarded. All following analyses were constructed on high quality reads.

### *De novo* transcriptome assembly and annotation

*De novo* transcriptome assembly of the RNA-Seq reads was performed using Trinity (version: r20140413p1) software to obtain high-quality transcript sequences [34]. The Trinity *in silico* normalization utility was used to normalize the large RNA-Seq data sets. Then, Trinity was employed for *de novo* assembly using paired-end RNA-Seq reads with the default parameters. Transdecoder was used for open reading frame (ORF) identification using the default parameters [34]. The Kyoto Encyclopedia of Genes and Genomes (KEGG) database was used for obtaining KEGG metabolic pathway data.

### GO enrichment analysis

To understand the biological functions of the differentially expression genes (DEGs), the assembled sequences were used for a homology search against the UniProt databases by ncbi-blast-2.2.27+ with an E-value of 10^−6^. The GO terms were assigned to assemble the sequences based on the UniProt databases (EMBL UniProt eggNOG/GO pathways databases). Then, all of the DEGs were mapped to the GO database and classified into three categories including “cellular component”, “molecular function” and “biological process”. The GO terms for DEGs were compared with the whole transcriptome background for GO enrichment analysis. GO enrichment analysis was performed on all DEGs using BINGO 3.0.2 [63] according to the custom GO annotation files from the transcriptome to identify the overrepresented GO terms in the DEGs. A *P* value cutoff of 0.01 (hypergeometric test with Benjamini and Hochberg false discovery rate correction) was used to determine the enriched GO terms.

### Gene expression analysis (GEA) and the identification of DEGs

GEA was performed in two sequential steps: (1) mapping all the clean reads to the assembled sequences using Bowtie2 2.2.3 [35] to calculate the read counts for each transcript and (2) measuring and normalizing the transcript abundances for each gene as the fragments per kilobase of exon per million fragments mapped (FPKM), which is analogous to single-read Reads Per Kilobase per Million mapped reads [64]. To more accurately calculate the expression level of the assembled genes, only both pairs of paired-end reads with unique location were retained to calculate expression values. We then calculated the *P* value using the MA-plot-based method with random sampling model in the DEGseq R package [36]. Then, genes with a fold change > 3 and a *P* < 0.001 were identified as DEGs. The hierarchical clustering of DEGs was performed using gplots in the R program environment using FPKM expression values in each RNA-Seq library.

### Validation of differential expression genes by qRT-PCR

Total RNAs as described for RNA-Seq library construction were used for qRT-PCR. Reverse transcription was performed using 1 μg total RNA for each sample and 200 U M-MLV Transcriptase (TaKaRa) in a 10 μl volume. The reaction was conducted at 70 °C for 10 min, 42 °C for 60 min and 70 °C for 15 min. The resulting cDNA was diluted to 800 μl with sterile water. The qRT-PCR was conducted in triplicate reactions using the BIO-RAD CFX system (BIO-RAD). Gene-specific primers were designed by Primer3 (http://bioinfo.ut.ee/primer3/), and the primers for randomly selected 28 genes used in this study are listed in Additional file 16. The primers for validation of all the genes that described in the result part are listed in Additional file 17.

The 40S ribosomal protein S8 (c42687_g2) was used as the internal control for qRT-PCR because the expression level of this gene is relatively stable in samples of different tissues and different time points. The PCR was conducted in a 20 μl volume containing 4 μl diluted cDNA, 250 nM forward primer, 250 nM reverse primer, and 1 × SYBR Premix Ex Taq II (TaKaRa) using the following conditions: 95 °C for 3 min, 40 cycles of 95 °C for 15 s, 59 °C for 15 s and 72 °C for 15 s. Melting curve analyses were performed to verify the specificity using the Bio-Rad CFX Manage software. The relative expression levels were calculated using the 2^-ΔΔCT^ method [65].

### Alternative splicing identification

Because the *P. ginseng* genome is currently unavailable, we followed previous methods to identify alternative splicing (AS) events to obtain AS events based on assembled transcript sequences [25]. In brief, the Trinity assembled sequences were compared with each other by BLAT with the following options: −tileSize = 18 -oneOff = 0 -minIdentity = 96 -out = sim4 -maxIntron = 10000. Finally, the candidates were identified according to the pair-wise comparisons of assembled transcripts from the same locus to search for indels as candidate regions. Indels that were flanked by two sequentially matching sections from the pair comparison of assembled transcripts were regarded as candidate AS regions.

### Validation of AS events by RT-PCR

Equal amounts of total RNA as described for RNA-Seq library construction were pooled and used for reverse transcription PCR (RT-PCR) to validate the AS events. Reverse transcription was carried out using 2 μg of total RNA by 200 U M-MLV Reverse Transcriptase (TaKaRa) in a 20 μl volume under the following the conditions: 65 °C for 5 min; 25 °C for 10 min; 42 °C for 60 min and 70 °C for 15 min. The resulting cDNA was used for PCR amplification, which was performed under the following conditions: 95 °C for 3 min; 45 cycles of 94 °C for 30 s; 58 °C for 30 s; and 72 °C for 15 s. The PCR products were separated by electrophoresis with a 3 % agarose gel. Primers used for the RT-PCR validation of AS events are listed in Additional file 15.

### Root morphology under benzoic acid treatment

After ripening, the ginseng seeds were sterilized by 50 % carbendazol wettable powder (diluted 800 times) for 10 min followed by rinsing three times in distilled water. Then, the seeds were cultivated in Petri dishes at 20 °C under dark conditions with five different concentrations of benzoic acid (0, 0.25, 2.5, 25, 250 mg · L^−1^). Seven days later, the root morphology was observed at 10 times magnification by LeicaS8APO stereomicroscope via the Anymicro DSS YT-7 M microscopic digital camera system. Ten ginseng seedlings were observed for each concentrations of benzoic acid. In total, 50 ginseng seedlings were investigated in this study.

### Measurement of physiological and biochemical indexes

The seedlings were cultivated in the same conditions as the sequencing materials. Then the seedlings were treated with four different concentrations of benzoic acid (0, 2.5, 25, 250 mg · L^−1^). The content of proline in the leaves was measured at 0, 1, 3, 5, 7 and 9 DPT using the ninhydrin method [66]. The MDA content was measured using the thiobarbituric acid method [67]. The content of both proline and MDA were measured with three biological replicates.

## Additional files

Additional file 1:
**Sequence length distribution of assembled transcripts.** The X-axis indicates the length range of the transcript sequences. The Y-axis indicates the percentage of transcript sequences with a certain length. (PDF 81 kb) Additional file 2:
**All the annotation information for the assembled transcripts.** (XLS 26730 kb) Additional file 3:
**Venn diagram showing annotated genes by SP, NR, NT, and KEGG.** The number of annotated genes is listed in each diagram component. (PDF 112 kb) Additional file 4:
**Plotting the distribution of log-transformed FPKM values across 18 libraries.** The X-axis indicates the log2 of the FPKM value, and the Y-axis indicates the percentage of genes with a given FPKM value. (PDF 241 kb) Additional file 5:
**The lists for all the differentially expressed genes.** (XLS 1362 kb) Additional file 6:
**The expression profiles based on RNA-Seq for 28 randomly chosen DEGs.** (JPEG 1560 kb) Additional file 7:
**The RNA-Seq profiles together with the qRT-PCR validations for DEGs across all the time points.** (TIFF 1778 kb) Additional file 8:
**The RNA-Seq profiles and qRT-PCR validations for the ROS-related genes.** (TIFF 1356 kb) Additional file 9:
**The RNA-Seq profiles and qRT-PCR validations for P5CS, P5CR and APX.** (TIFF 747 kb) Additional file 10:
**The RNA-Seq profiles and qRT-PCR validations for root hair related genes.** (TIFF 2900 kb) Additional file 11:
**The RNA-Seq profiles and qRT-PCR validations for genes involved in flavonoid and ginsenoside biosynthetic pathways.** (TIFF 1953 kb) Additional file 12:
**The RNA-Seq profiles and qRT-PCR validations for TFs upon benzoic acid Treatment.** (TIFF 2528 kb) Additional file 13:
**The differentially expressed genes across all the time points.** (XLS 66 kb) Additional file 14:
**Benzoic acid inhibited the growth of root hairs.** (JPEG 1687 kb) Additional file 15:
**Primers of 17 genes for alternative splicing validation.** (PDF 13 kb) Additional file 16:
**Primers of 28 randomly chosen DEGs for validation.** (PDF 506 kb) Additional file 17:
**Primers of all the genes described in the result section for qRT-PCR validation.** (PDF 91 kb)

## Abbreviations

ANS: anthocyanidin synthase
AP2: APETALA 2 transcription factor
APX: ascorbate peroxidase
AS: alternative splicing
A3GT: anthocyanidin 3-O-glucosyltransferase
BLAST: basic local alignment search tool
CAT: Catalase
CHS: chalcone synthase
CHI: chalcone isomerase
cDNA: complementary DNA
DCF: dihydrodichlorofluorescein
DEG: differentially expressed gene
DPI: day post-inoculation
ERF: ethylene responsive factor
EMBL: European Molecular Biology Laboratory
FPKM: fragments per kilobase of exon per million fragment
F3H: flavanone 3-hydroxylase
F3’5’H: Flavonoid 3’ 5’-hydroxylase
F3’H: Flavonoid 3’-hydroxylase
FA: Ferulic acid
GO: gene ontology
GEA: gene expression analysis
KEGG: kyoto encyclopedia of genes and genomes
MDA: malondialdehyde
MYB: transcription factor containing a Myb DNA binding domain
NAM: no apical meristem
NR: non-redundant protein database
NT: nucleotide database
ORF: open reading frame
POD: peroxidase
RNA-Seq: RNA sequencing
RT-PCR: reverse transcription-PCR
RPKM: Reads Per Kilobase per Million mapped reads
ROS: Reactive oxygen species
RIN: RNA integrity number
SOD: superoxide dismutase
STEM: short time-series expression miner
WRKY: (WRKYGQK)
TF: transcription factor
