# Supplementary figures and images for: Comprehensive characterization of a time-course transcriptional response induced by autotoxins in Panax ginseng using RNA-Seq
Source: BMC Genomics. 2015 Nov 25;16:1010. doi: 10.1186/s12864-015-2151-7 (PMC4659204; doi:10.1186/s12864-015-2151-7)

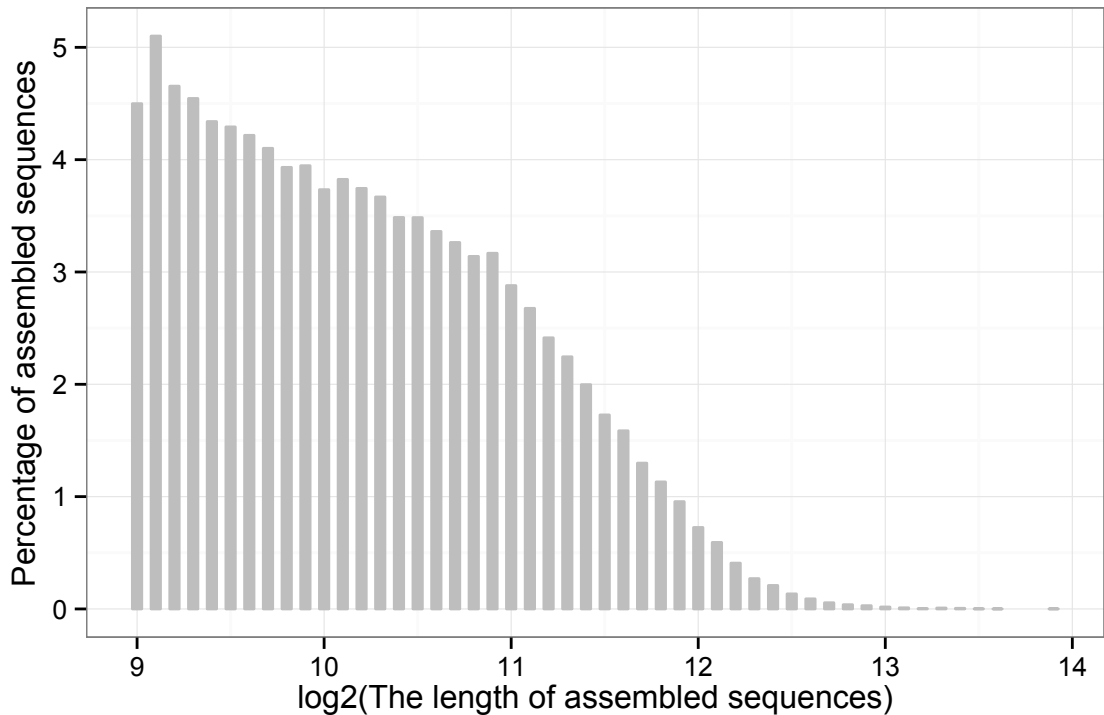

Supplement: Additional file 1: — Sequence length distribution of assembled transcripts. The X-axis indicates the length range of the transcript sequences. The Y-axis indicates the percentage of transcript sequences with a certain length. (PDF 81 kb) [file 12864_2015_2151_MOESM1_ESM.pdf]

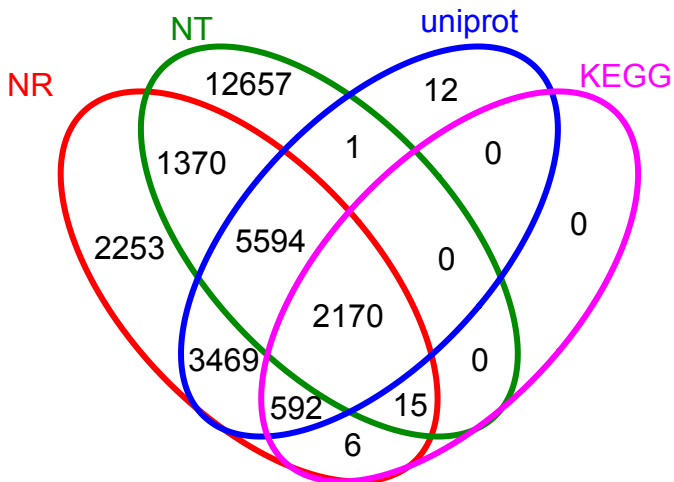

Supplement: Additional file 3: — Venn diagram showing annotated genes by SP, NR, NT, and KEGG. The number of annotated genes is listed in each diagram component. (PDF 112 kb) [file 12864_2015_2151_MOESM3_ESM.pdf]

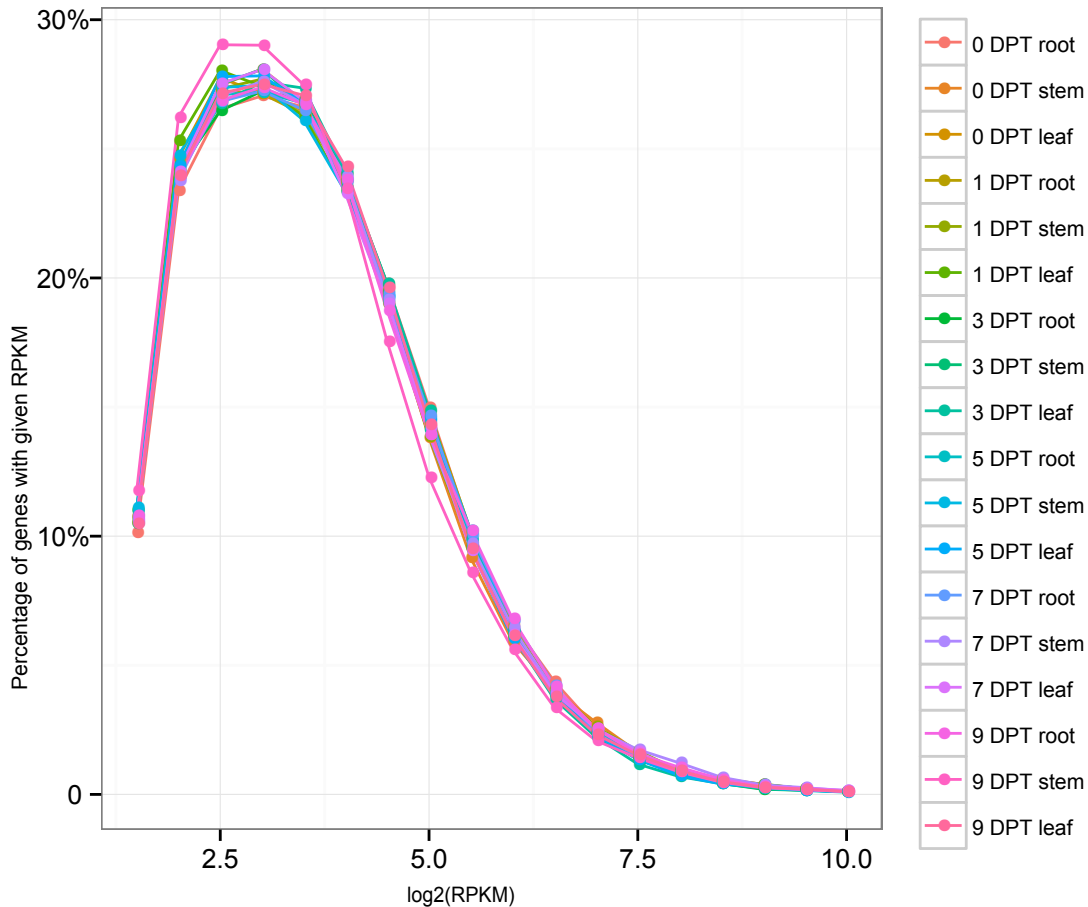

Supplement: Additional file 4: — Plotting the distribution of log-transformed FPKM values across 18 libraries. The X-axis indicates the log2 of the FPKM value, and the Y-axis indicates the percentage of genes with a given FPKM value. (PDF 241 kb) [file 12864_2015_2151_MOESM4_ESM.pdf]

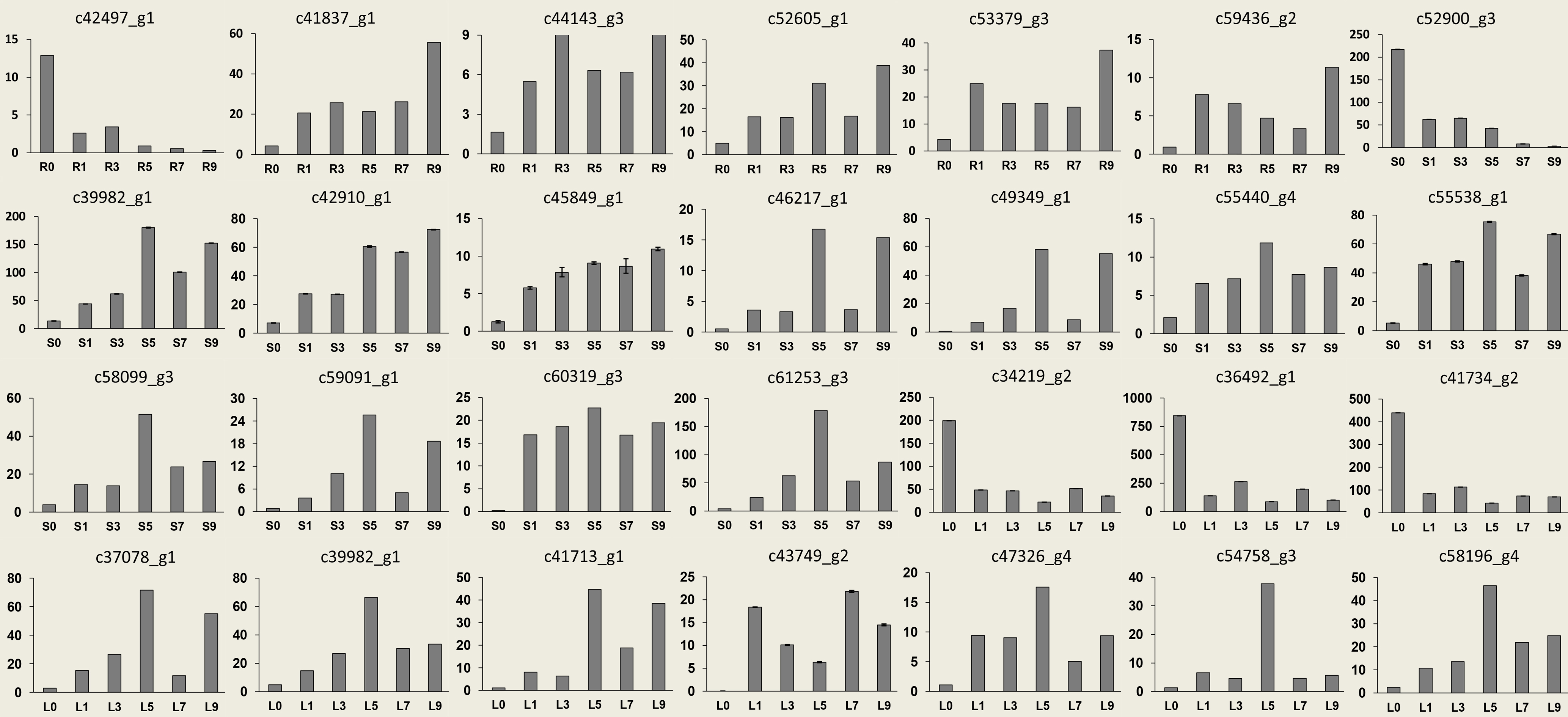

Supplement: Additional file 6: — The expression profiles based on RNA-Seq for 28 randomly chosen DEGs. (JPEG 1560 kb) [file 12864_2015_2151_MOESM6_ESM.jpg]

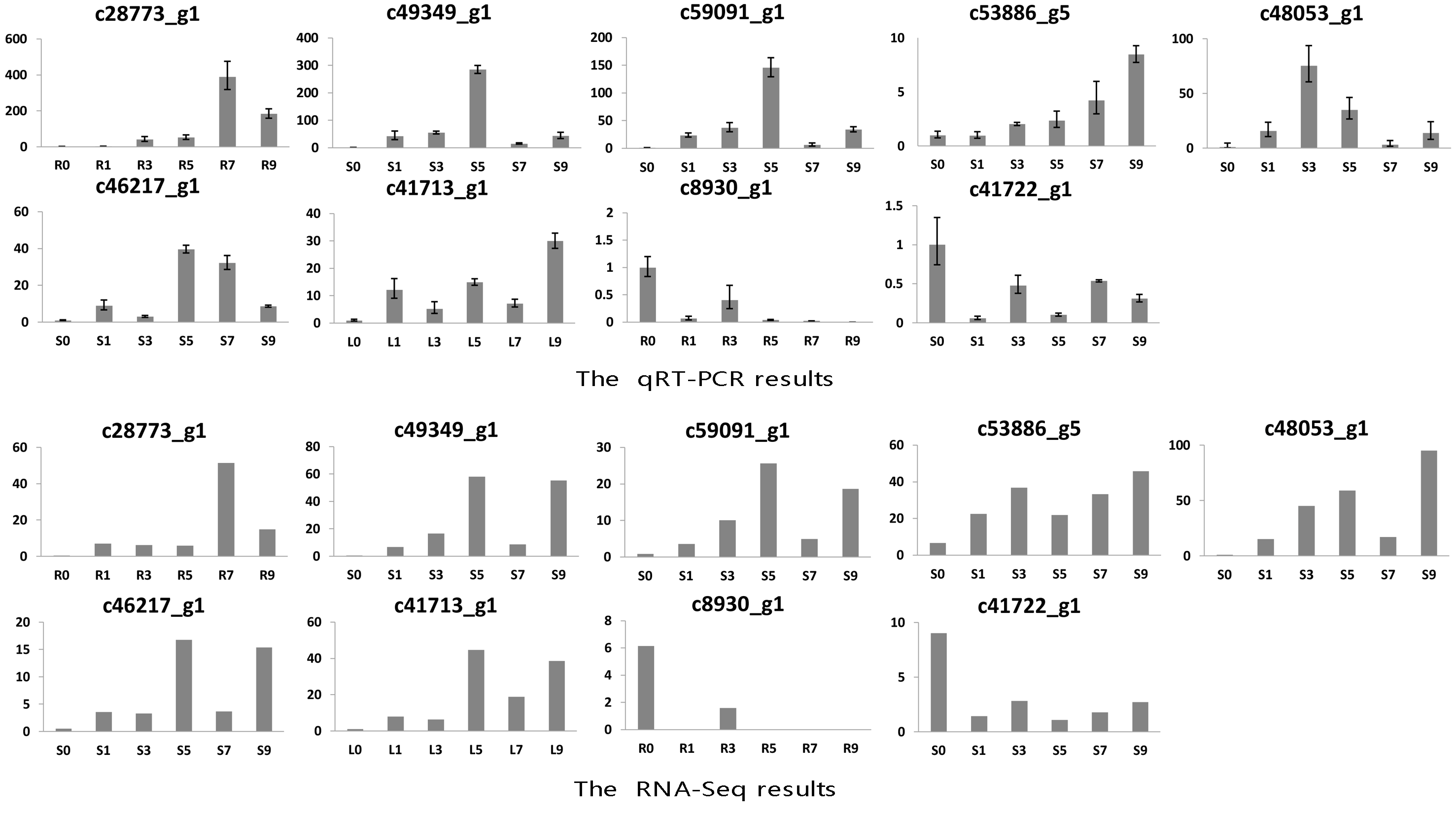

Supplement: Additional file 7: — The RNA-Seq profiles together with the qRT-PCR validations for DEGs across all the time points. (TIFF 1778 kb) [file 12864_2015_2151_MOESM7_ESM.tif]

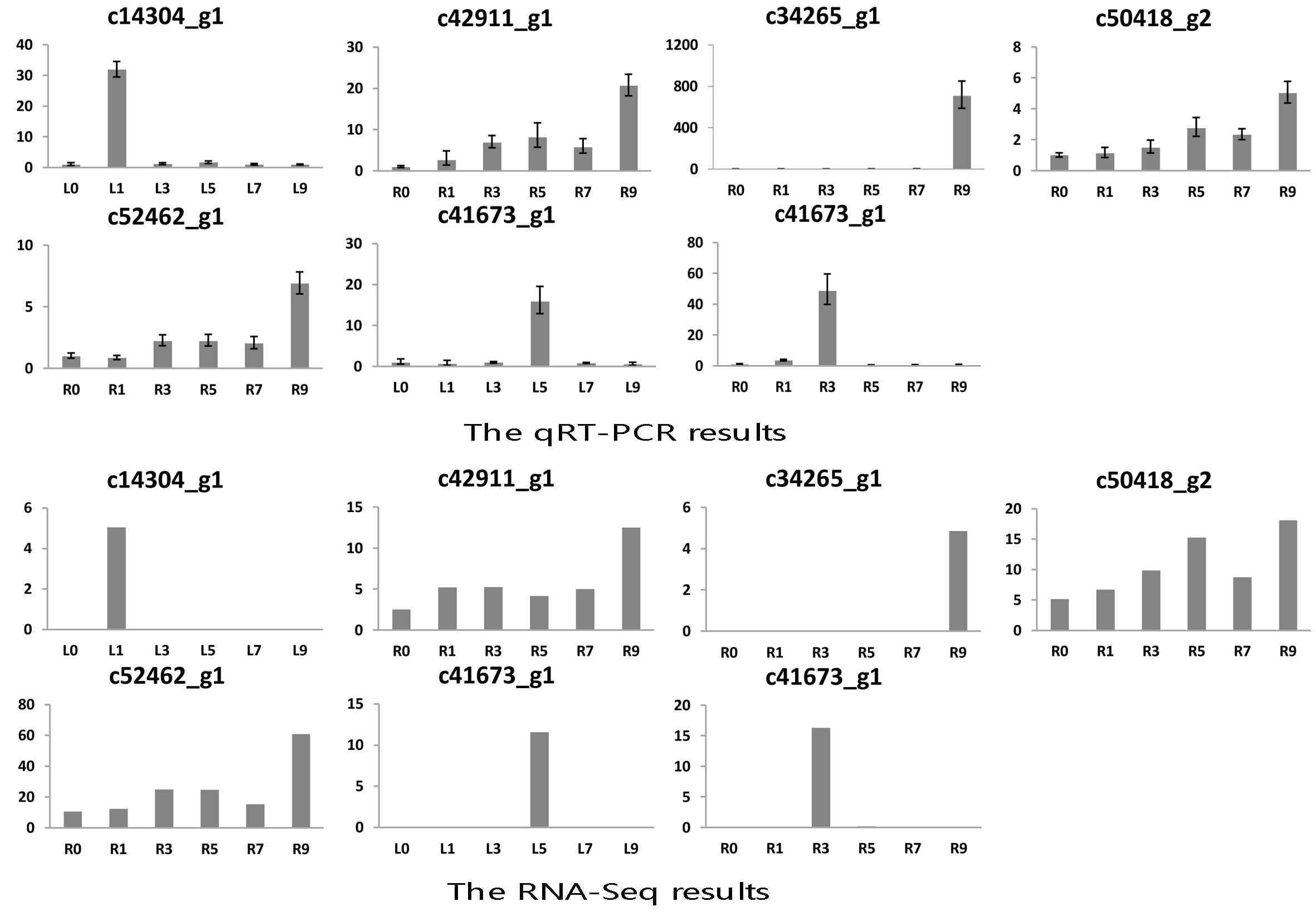

Supplement: Additional file 8: — The RNA-Seq profiles and qRT-PCR validations for the ROS-related genes. (TIFF 1356 kb) [file 12864_2015_2151_MOESM8_ESM.tif]

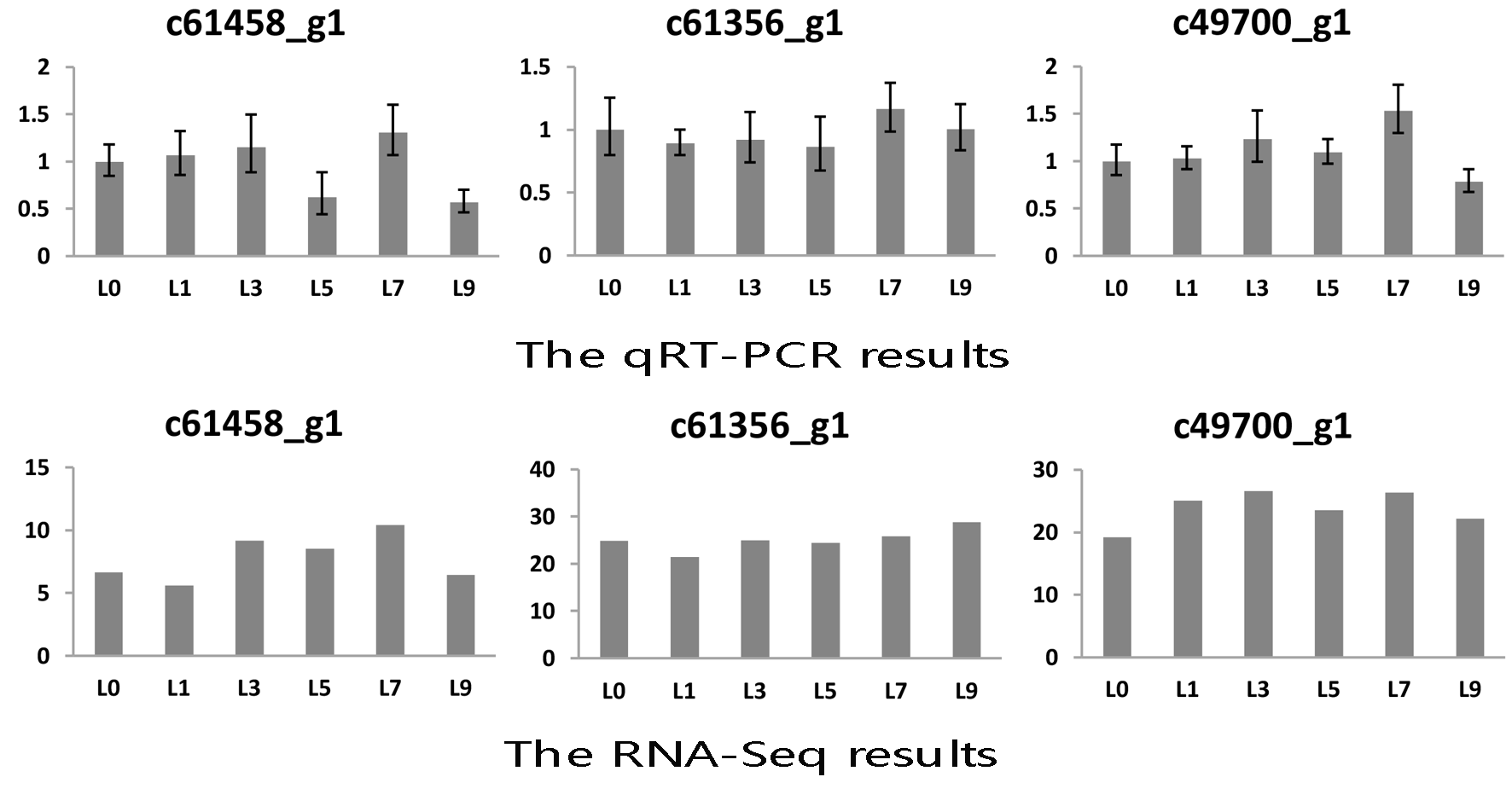

Supplement: Additional file 9: — The RNA-Seq profiles and qRT-PCR validations for P5CS, P5CR and APX. (TIFF 747 kb) [file 12864_2015_2151_MOESM9_ESM.tif]

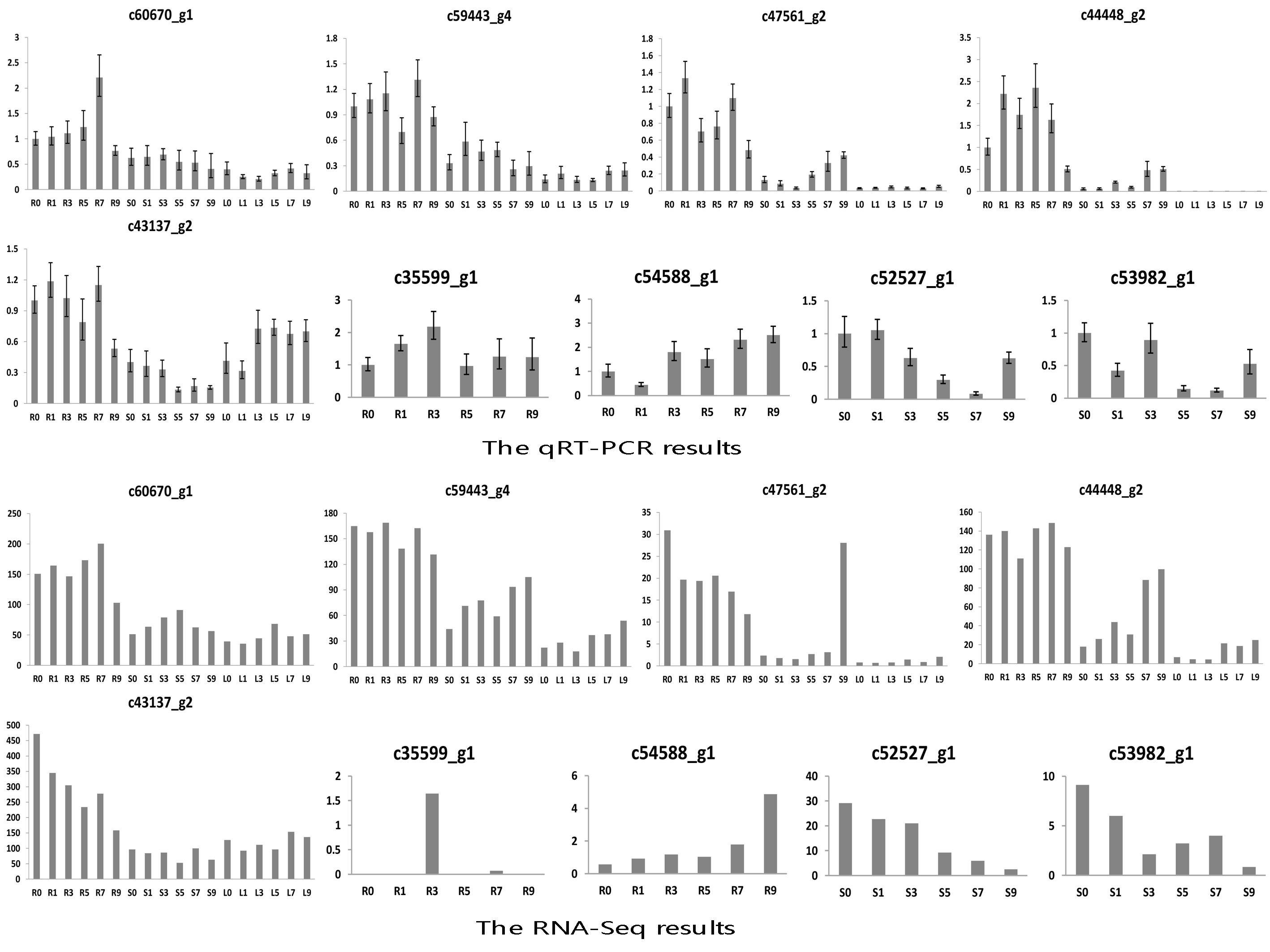

Supplement: Additional file 10: — The RNA-Seq profiles and qRT-PCR validations for root hair related genes. (TIFF 2900 kb) [file 12864_2015_2151_MOESM10_ESM.tif]

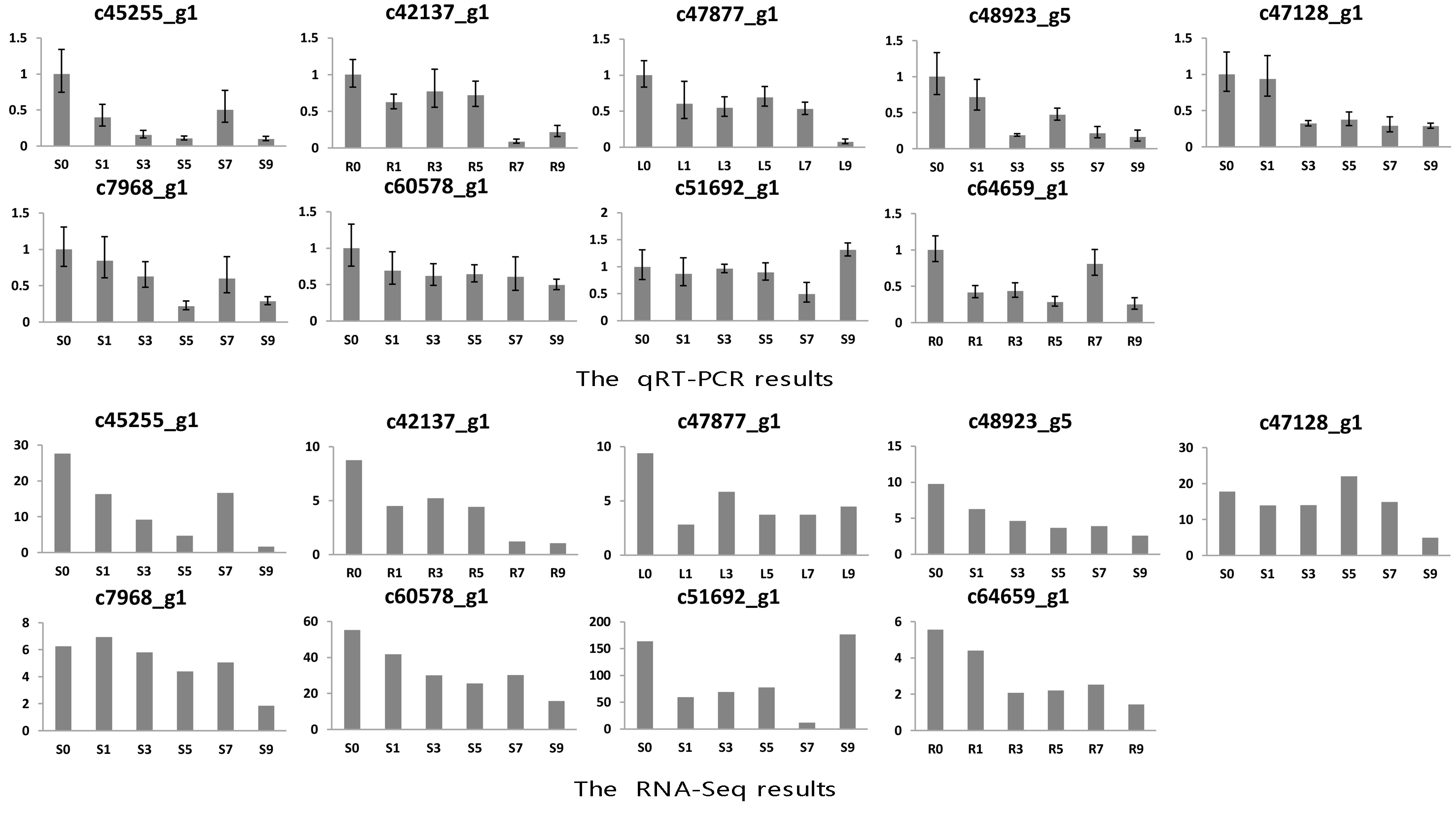

Supplement: Additional file 11: — The RNA-Seq profiles and qRT-PCR validations for genes involved in flavonoid and ginsenoside biosynthetic pathways. (TIFF 1953 kb) [file 12864_2015_2151_MOESM11_ESM.tif]

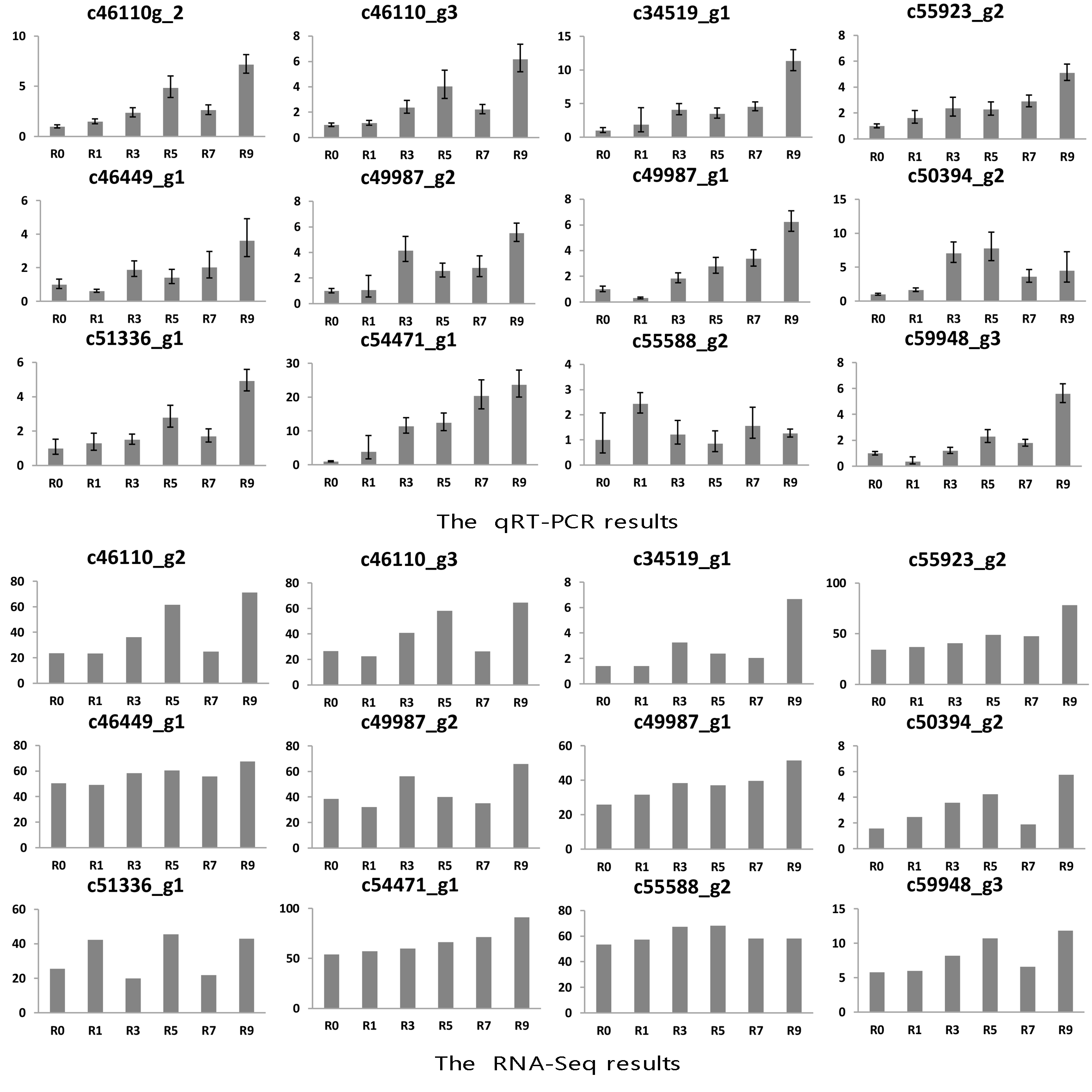

Supplement: Additional file 12: — The RNA-Seq profiles and qRT-PCR validations for TFs upon benzoic acid Treatment. (TIFF 2528 kb) [file 12864_2015_2151_MOESM12_ESM.tif]

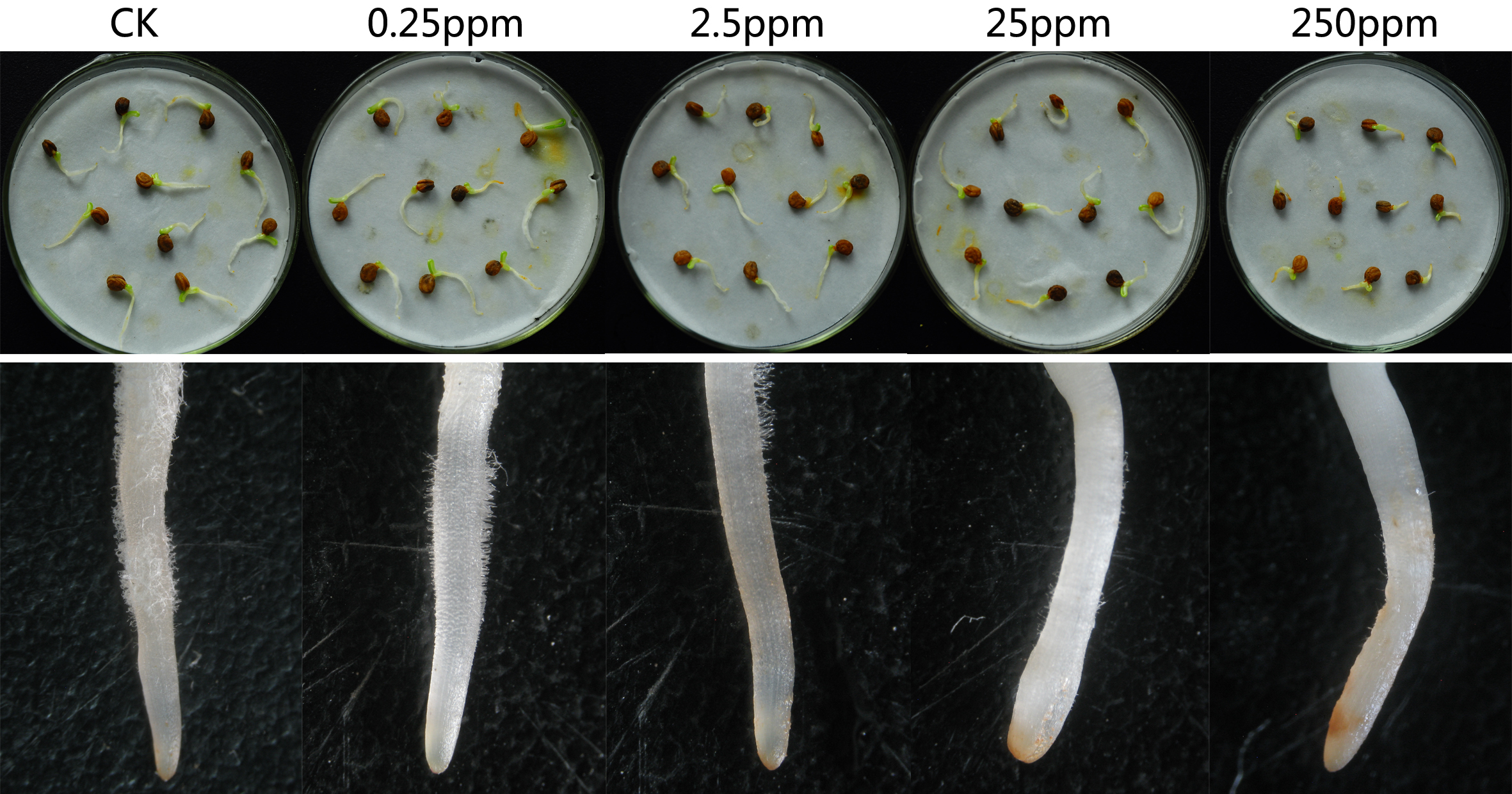

Supplement: Additional file 14: — Benzoic acid inhibited the growth of root hairs. (JPEG 1687 kb) [file 12864_2015_2151_MOESM14_ESM.jpg]
