# Supplementary material for: Comprehensive characterization of a time-course transcriptional response induced by autotoxins in Panax ginseng using RNA-Seq
Source: BMC Genomics. 2015 Nov 25;16:1010. doi: 10.1186/s12864-015-2151-7 (PMC4659204; doi:10.1186/s12864-015-2151-7)
Supplement: Additional file 15: — Primers of 17 genes for alternative splicing validation. (PDF 13 kb) [file 12864_2015_2151_MOESM15_ESM.pdf]

Primers of 17 genes for alternative splicing validation

Contig Name	Primer sequences (5'-3')	PCR products size (bp)		Verification
c46446_g3	CGGAATACATTTGTGGATGGCT	321	189	Yes
	AATGGTCAGGTCGTTGTTCC			
c40764_g1	AATCCATGCACAAACACCCC	408	316	Yes
	GCGAGCATTGATTTGGAGGT			
c44919_g6	ACTTGCGCTGAGGTAGGTTA	353	199	Yes
	AGCACCCTGAGTTCTGAAGA			
c42192_g	CACCAAAGGAGGGCGAATAT	303	205	Yes
	ACACAACAAACTCTGCAGCA			
c27214_g1	GAATAGACGTTTGCTCCCGC	320	171	Yes
	GAATGGCTAGTTGGGCTTCC			
c61203_g1	ATCATCCTTTCGCGCTCATC	414	236	Yes
	AACACCCTCTCACCCTCATC			
c49302_g4	CAAATATGGAGGTGGCAGTCG	316	220	Yes
	TGCAGACTCCATGAAGCAAG			
c15452_g1	GTACCGGAATCAATGGCACC	313	196	No
	GCCCGAGCCATTGTAACATT			
c34793_g1	AAAGGGAGGCCAAACGTAGA	341	236	Yes
	ACCCCATGCCAAATACAGAA			
c15184_g1	CCCAATCCTAGTCGACCTCC	405	315	Yes
	TGACCCACATCGTAGGAACA			
c42813_g1	GAAGGGGTGAGGCTAGAAGG	337	222	Yes
	TGATCCGGGCTTTGAAGAGA			
c56267_g8	GGGGTAGAGGTTGAGGGTTG	367	189	Yes
	AATCTCCCGGGGTTGTCTTC			
c50955_g3	CAAACAGTGAGCCGTCGATT	343	250	Yes
	GACAGTGGAGTTTTCGGCAG			
c34239_g1	ATGGCGGTTCTTGGAATTCC	316	231	Yes
	CCCTGTTTGTTTGATCCGCA			
c5156_g1	CCACCGTTTCTTCCTCCTCT	338	224	Yes
	CTTCACCTCCTTCACTCCGT			
c11325_g1	CTGACTGTGTTGTTGCTCCC	358	235	No
	GCACATGGAGTTCTGTTGGG			
c62330_g2	TGGGGCTAGCATGATAACTGT	300	168	Yes
	ATGAGAGGTTACGCAGGCTC			
