# Supplementary material for: Comprehensive characterization of a time-course transcriptional response induced by autotoxins in Panax ginseng using RNA-Seq
Source: BMC Genomics. 2015 Nov 25;16:1010. doi: 10.1186/s12864-015-2151-7 (PMC4659204; doi:10.1186/s12864-015-2151-7)
Supplement: Additional file 16: — Primers of 28 randomly chosen DEGs for validation. (PDF 506 kb) [file 12864_2015_2151_MOESM16_ESM.pdf]

Genes	Primers	Sequences
c42497_g1	Forward	GGCACAAATAGCAGGACCTC
	Reverse	CTTGTGCTCCGCTTCTCTTG
c41837_g1	Forward	GATTCCCTGAGCATGCCTTG
	Reverse	AGAGCAAGCACCATTCTCCT
c44143_g3	Forward	AGGGCCGTTTCGAATCAGAA
	Reverse	GGGTTTGTTTCGCCAAGTCA
c52605_g1	Forward	GTAAAAGCTCCTATGGCGCC
	Reverse	AGGGGTCCACTCTTTTCAGC
c53379_g3	Forward	GTTCCAGGTCGCTTGTCATC
	Reverse	CGAGCTTCACAGAATGCCTC
c59436_g2	Forward	GCCACTGAGTTCTTGAACCC
	Reverse	GCAGGAGGAGTGGAGGTATT
c52900_g3	Forward	CCTCGTCCCTGAAGTATCGA
	Reverse	AGTGGGATAAGCACGGGAAA
c39982_g1	Forward	TCTCATCCCGATTAGCACCC
	Reverse	TTGGCTTGGTTTGGCGTATT
c42910_g1	Forward	GTGTATGGCATGGGAGGAGA
	Reverse	GAAAATGGAGTCGAACCGCA
c45849_g1	Forward	ACTCTGTTTTGGTGTTTTGGGT
	Reverse	TTAAAACCCAGGAAACCCGG
c46217_g1	Forward	CCACCAACCACAACATCAGG
	Reverse	GCACCATCTTCATCTGCTGG
c49349_g1	Forward	TGATGATGGACTTACGGCGA
	Reverse	TTCAATCCTGCACAACGAGC
c55440_g4	Forward	ACCAGAAGCAGAGTGAAGACA
	Reverse	AGGAAAGGAGACATGCCCAA
c55538_g1	Forward	GAGTAGTGATGGCAATGGAGC
	Reverse	GGGAGGTTTAAGAGTGTAAGGC
c58099_g3	Forward	TCCGCCTCAATCCATAGCAT
	Reverse	GGGATGTGAAACGATGGAGC
c59091_g1	Forward	AGAATCTCAGAGCAGCCAGA
	Reverse	ACTGGCATGTCTAACTGGCT
c60319_g3	Forward	CACCAATTCACCGCCAATGA
	Reverse	CTGCGGTTTATGAGCTCGAC
c61253_g3	Forward	ACGATCAGGACACATGCATT
	Reverse	CGACGACTGATGATGATGTGT
c34219_g2	Forward	GTGCCTGTTTCGTCACACAA
	Reverse	CCCACTTGCCACACTTTGAA
c36492_g1	Forward	ACAAAGCAATCAAGGCCTCG
	Reverse	AACGCGTTCAAAACCAGTGA

c41734_g2	Forward	CCGGCCATGCAAAATATCCA
	Reverse	GGAGGAGAGGAATTAGGGCC
c37078_g1	Forward	TCATTCGTTCATTCATGGCGT
	Reverse	ATCTCGAGCACTGTCCAAAA
c41713_g1	Forward	CTCAGCAGAAAAGGCAAGCA
	Reverse	GGGCTTGTTTGGATGCTACT
c43749_g2	Forward	TTTCACTCAGACCCCAACCA
	Reverse	TTGGGTGGCGTTGTTAATGG
c47326_g4	Forward	AACCTACTGCACCTGAAGCT
	Reverse	AGGCTCAAGAACGGACTCAT
c54758_g3	Forward	CCATGCTCAAAACTCCCCAC
	Reverse	TGGCGTAGTTTTGTGCGTAG
c58196_g4	Forward	AGCTCAAACGACTATATGCGT
	Reverse	TATCCGACAAGACACAGGGG
c42687_g2	Forward	TGTTCAGGTTGATGCTGCAC
	Reverse	TCGGTAGGAGTGTCAGCATC
