# Supplementary material for: Comprehensive characterization of a time-course transcriptional response induced by autotoxins in Panax ginseng using RNA-Seq
Source: BMC Genomics. 2015 Nov 25;16:1010. doi: 10.1186/s12864-015-2151-7 (PMC4659204; doi:10.1186/s12864-015-2151-7)
Supplement: Additional file 17: — Primers of all the genes described in the result section for qRT-PCR validation. (PDF 91 kb) [file 12864_2015_2151_MOESM17_ESM.pdf]

Genes	Primers	Sequences
c28773_g1	Forward	GGGACAGAGCGTTGATGTTC
	Reverse	CGATCCTGATTTTGCCAGCA
c53886_g5	Forward	CTGCCCTCGACTATCAGCTT
	Reverse	CTCACACGTGCACACTCATT
c48053_g1	Forward	GCGACGGACAAATGATGGAA
	Reverse	GGCTCAAATAGTCCAGCTGC
c8930_g1	Forward	CGAGGCCATTGACTTCTTCG
	Reverse	TATCCAAGACATAGCGGCCG
c41722_g1	Forward	AAGAGAATGCTGAGCCGACT
	Reverse	CAACGCATCATGAGCCTGAA
c14304_g1	Forward	TTTGAAGTGCCCTTGTTCGG
	Reverse	ATTCAGCTTTCATTGGGCCG
c42911_g1	Forward	CCATGGACAGGAGCAAAAGT
	Reverse	CCTCCAACAAAGGCACATCC
c34265_g1	Forward	TTCGGTGGAAAGTGTGCAAC
	Reverse	ACAGCAGTCCCATTAGCGAT
c50418_g2	Forward	ACCAACCCTACAGAAGCCTC
	Reverse	AACATTGTGCCTGCCCAAAT
c52462_g1	Forward	AGTGATCCCAAGTGCAGGAA
	Reverse	CCCATCTCGTCTTCCCAGTT
c41673_g1	Forward	AGCATGCCTACTACCCACAG
	Reverse	ATCACGGCTATGCTTCTCGA
c61458_g1	Forward	AGTCGAACTTGAGCAGCCTT
	Reverse	CGATCCGAACACCCTAATGC
c61356_g1	Forward	GGGAATCGATGACATGGCAC
	Reverse	ACATTGGGTGGCCTAGCATA
c49700_g1	Forward	CTCCCTCACCTCCTACTCCT
	Reverse	AAGCGGTGGATTGTGAGAGA
c60670_g1	Forward	GGAGCTCACTTAATGCCAGC
	Reverse	TAAGATCCAGAGCACGCACA
c59443_g4	Forward	TCCCTCTTACACAGGCTTGG
	Reverse	GTTGTCCAAGCTCAAACCCA
c47561_g2	Forward	TACAGGGCCATCATTGCAGA
	Reverse	AGCAATGCCAAGAATGCCAA
c44448_g2	Forward	TGGAATGGAAGTCGCAGGAA
	Reverse	ACGATCCGCAATACCAGGAA
c43137_g2	Forward	AGGCTCTTGGATCCTTCAGG
	Reverse	TCATAGACGACGACAGAGCA
c35599_g1	Forward	TTGTTGGGGAGGGTATGCAT
	Reverse	TCTCTCAGCACTTGACCTCC
c54588_g1	Forward	TAAACCAACACAAAGCGGGG

	Reverse	ATTTCTGTGTCAGCGGGGTA
c52527_g1	Forward	GCCATGCCATGAACTGACAT
	Reverse	AGTCCAAGTGCTCTGCTTCT
c53982_g1	Forward	ACATCCACACCCTTCTCCTG
	Reverse	CGACAAATGAACACGACCGT
c45255_g1	Forward	ACACATTCTCTTGAGGACGGA
	Reverse	GGCCTCCCATCCCCTATTTA
c42137_g1	Forward	GTTTAACGTCGATGCTCCCC
	Reverse	TGTTCACCATAGCTCGGTGT
c47877_g1	Forward	GGGGACATACTTTCAAGCACA
	Reverse	TACCCCGACTAGATCCTGCT
c48923_g5	Forward	TGAATCGAGGGCTGTCTGTT
	Reverse	AGCCATTCAATGCACCAACC
c7968_g1	Forward	CGATTGGTGGAAATGGGCTT
	Reverse	ACAACAGCAGCCTCCAAAAG
c47128_g1	Forward	AGAGCTCCTTACACCAGACG
	Reverse	TAGAGCCCATCCACCAATCC
c60578_g1	Forward	ACAAGCAAGGACTGTAGCCT
	Reverse	TTGCCAACGTTCCATTCTCG
c51692_g1	Forward	GGTGGGGATTAGGGTTAGGG
	Reverse	AGCCTCTTTAGCCAAACCCT
c64659_g1	Forward	AGGGAAGAGATGGGTGAACG
	Reverse	ATACCGGCACTTCACTCCAA
c46110_g2	Forward	CTTACAGGCCGGTGACATTG
	Reverse	AACGCACCAAGAACCGTAAC
c46110_g3	Forward	ACTTCCAAAGGCGTGCTTTT
	Reverse	CGGCCTTTAAGCACTTCTCC
c34519_g1	Forward	GCTACCGGGTCGAACTGATA
	Reverse	TTCTTTGGCTCTTGTCGCAC
c55923_g2	Forward	CGAGGAATCAGGAGAAGGCT
	Reverse	CCATTCCAAACCCAATCGCA
c46449_g1	Forward	GCTTTTCAAACGAGGAGCCA
	Reverse	CATTGCACCCTTGATGCGTA
c49987_g2	Forward	CCCTAGAGATGCTGCAAGGT
	Reverse	CTTGGCCTCAGAGAATTGCC
c49987_g1	Forward	CGGAGAGAAAGAGGTGGTCA
	Reverse	ATTTGGTTGGCAAGGCAAAA
c50394_g2	Forward	AGGAGTGCACAACCATGAGA
	Reverse	GAGCTTAAGATCCGGACCGT
c51336_g1	Forward	TCTGAGCTGTTGTCTCCGAC
	Reverse	AATGTACACCTCCCACTCCC
c54471_g1	Forward	CCTACCCCAATTGCCCAATG
	Reverse	GACTGAGGCTTGGATGAGT

c55588_g2	Forward	GGCAATTCGGGTGTCATGTT
	Reverse	CACTCACCCTTTCAACTCGC
c59948_g3	Forward	ATCCACAGCATCAGTCTCCC
	Reverse	GCCTGTTCCAAAGTTGAGCA
